# Supplementary figures and images for: Mesenchymal stem cells alleviate sepsis-induced acute lung injury by blocking neutrophil extracellular traps formation and inhibiting ferroptosis in rats (part 1 of 2)
Source: PeerJ. 2024 Jan 29;12:e16748. doi: 10.7717/peerj.16748 (PMC10832623; doi:10.7717/peerj.16748)

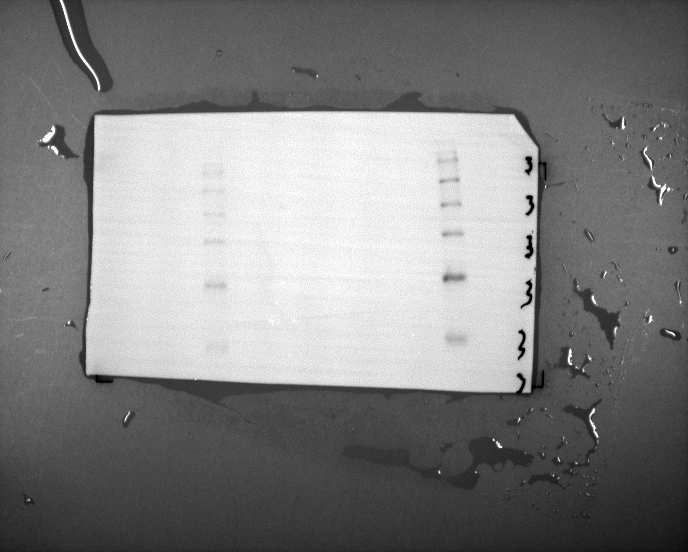

Supplement: Supplemental Information 9 [file peerj-12-16748-s009.zip › Figure 1,WB/3.tif]

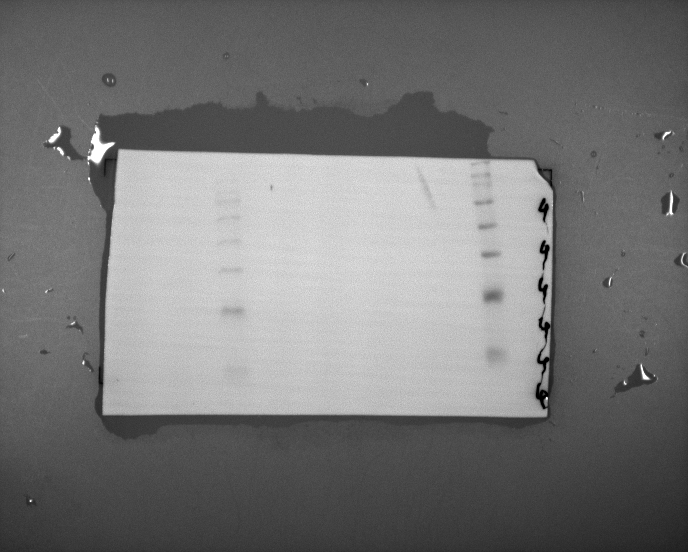

Supplement: Supplemental Information 9 [file peerj-12-16748-s009.zip › Figure 1,WB/4.tif]

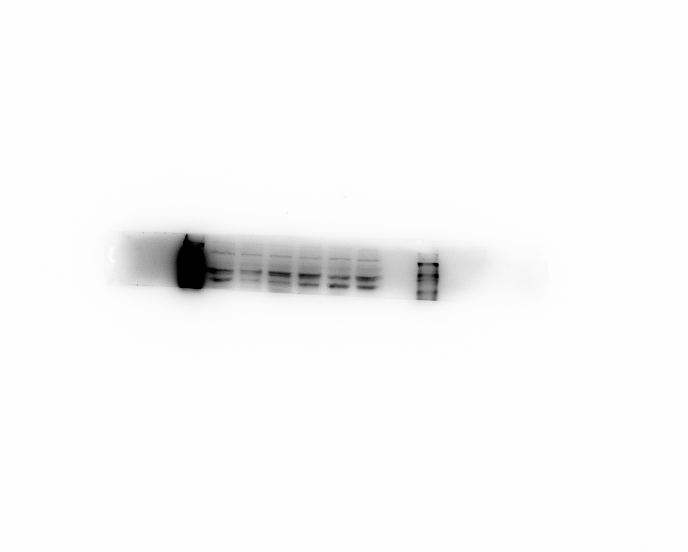

Supplement: Supplemental Information 9 [file peerj-12-16748-s009.zip › Figure 1,WB/acsl43-1.tif]

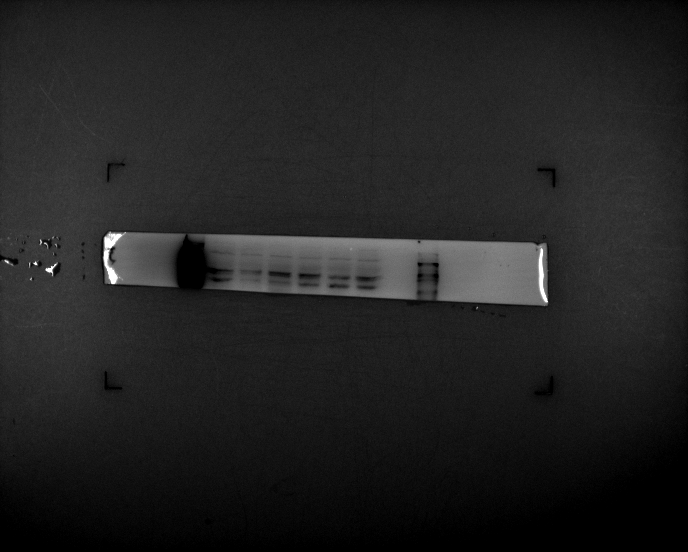

Supplement: Supplemental Information 9 [file peerj-12-16748-s009.zip › Figure 1,WB/acsl43-2.tif]

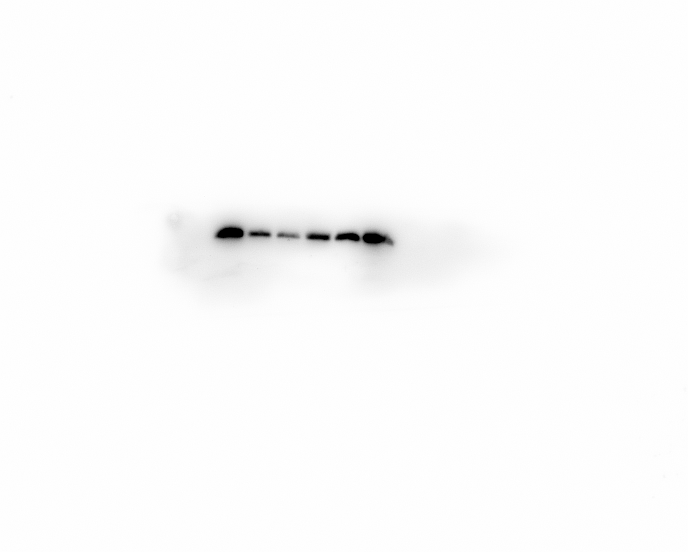

Supplement: Supplemental Information 9 [file peerj-12-16748-s009.zip › Figure 1,WB/fth3-1.tif]

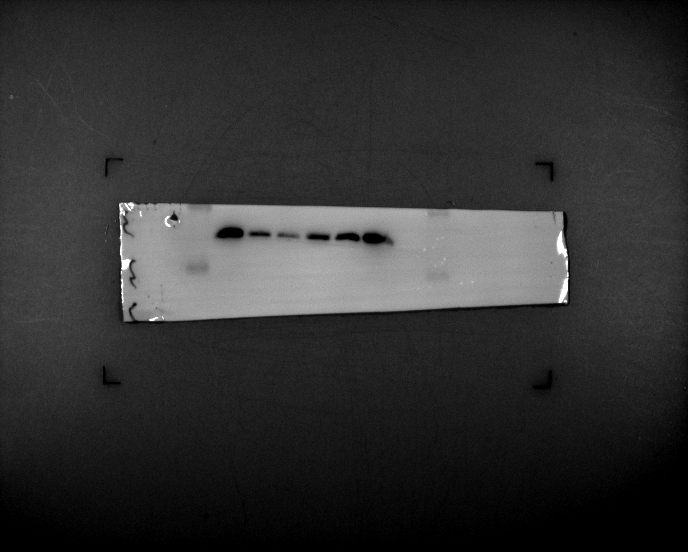

Supplement: Supplemental Information 9 [file peerj-12-16748-s009.zip › Figure 1,WB/fth3-2.tif]

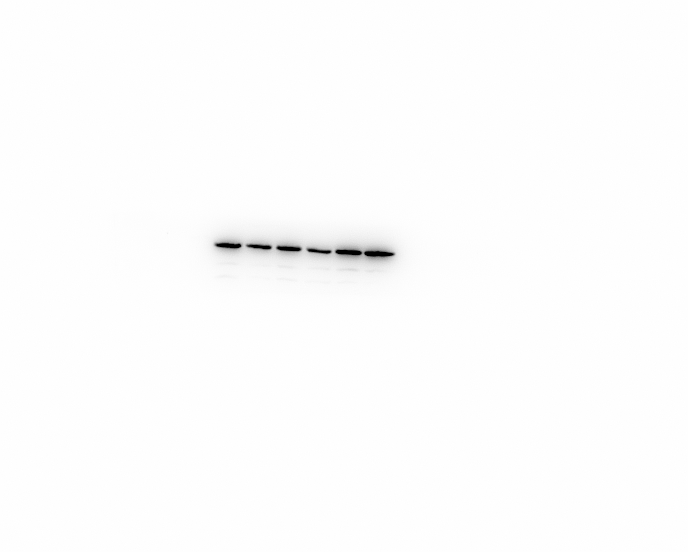

Supplement: Supplemental Information 9 [file peerj-12-16748-s009.zip › Figure 1,WB/gapdh3-1.tif]

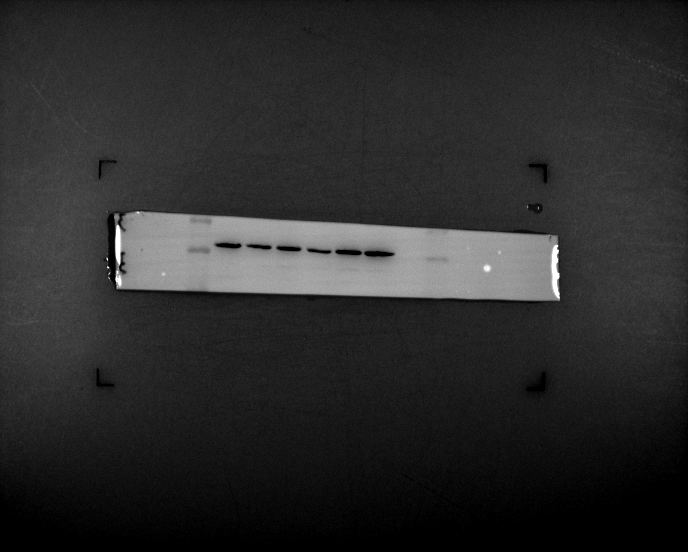

Supplement: Supplemental Information 9 [file peerj-12-16748-s009.zip › Figure 1,WB/gapdh3-2.tif]

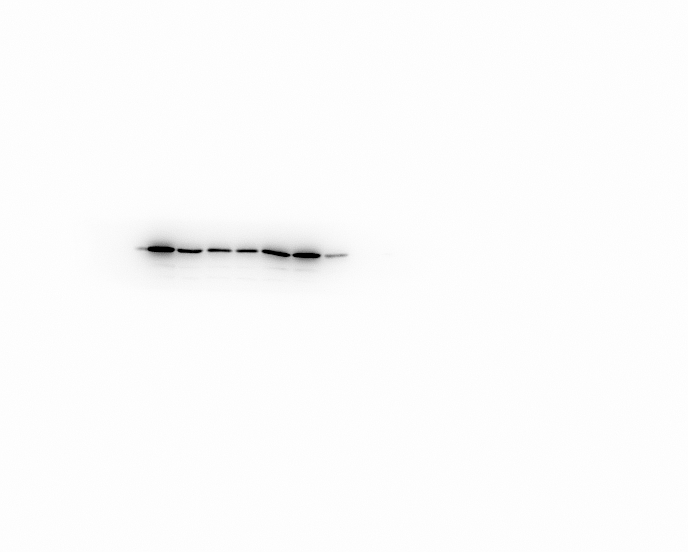

Supplement: Supplemental Information 9 [file peerj-12-16748-s009.zip › Figure 1,WB/gapdh4-1.tif]

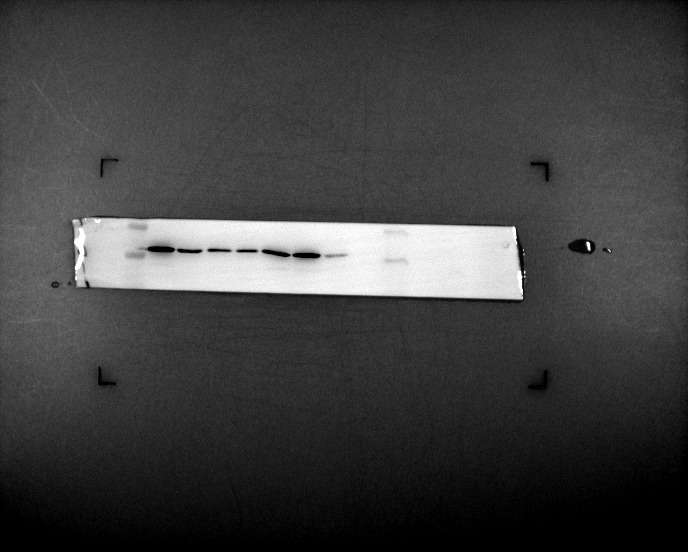

Supplement: Supplemental Information 9 [file peerj-12-16748-s009.zip › Figure 1,WB/gapdh4-2.tif]

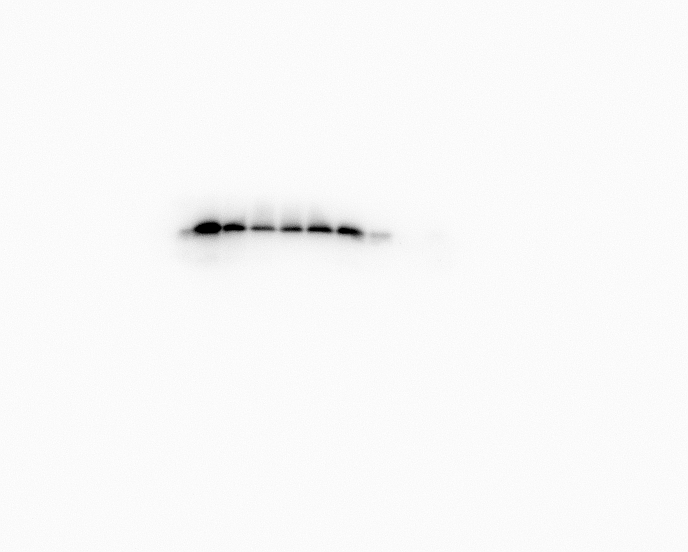

Supplement: Supplemental Information 9 [file peerj-12-16748-s009.zip › Figure 1,WB/gpx4-1.tif]

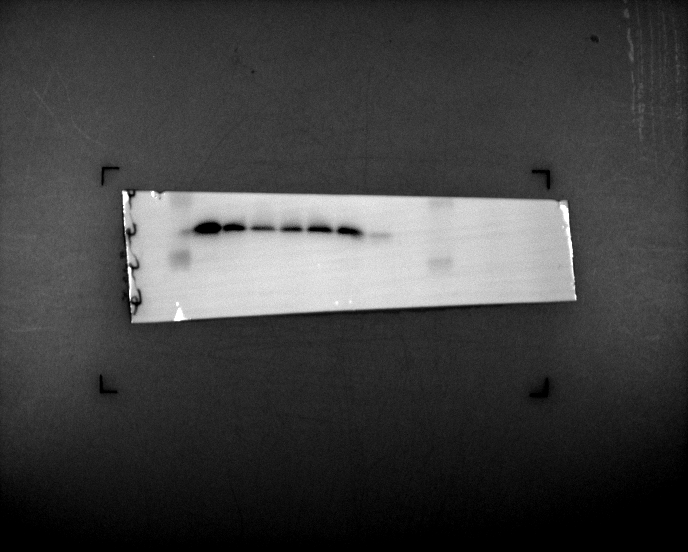

Supplement: Supplemental Information 9 [file peerj-12-16748-s009.zip › Figure 1,WB/gpx4-2.tif]

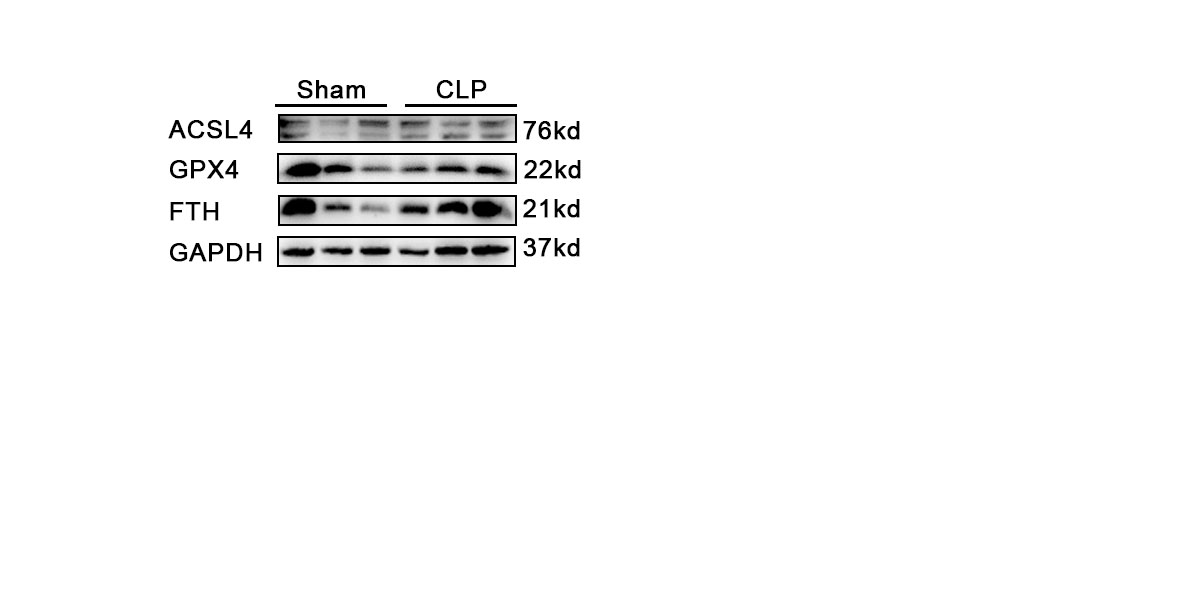

Supplement: Supplemental Information 9 [file peerj-12-16748-s009.zip › Figure 1,WB/WB-1.jpg]

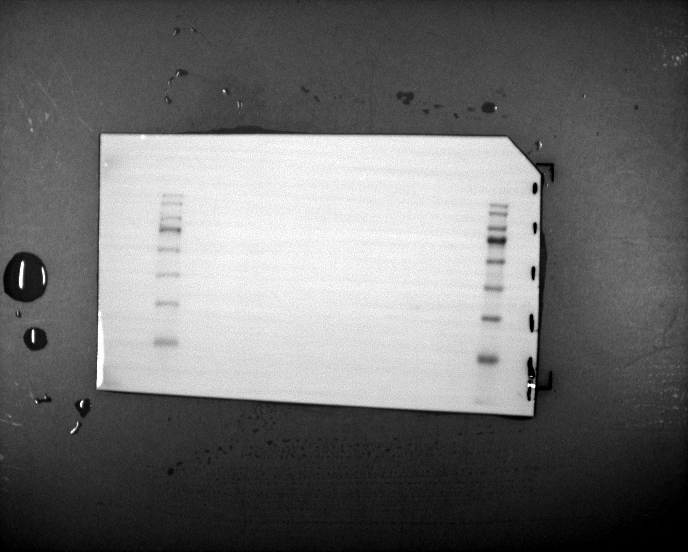

Supplement: Supplemental Information 10 [file peerj-12-16748-s010.zip › 1.tif]

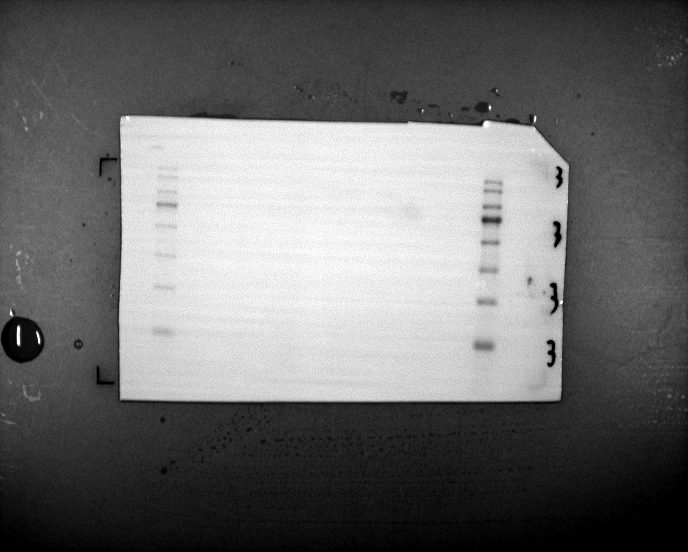

Supplement: Supplemental Information 10 [file peerj-12-16748-s010.zip › 3.tif]

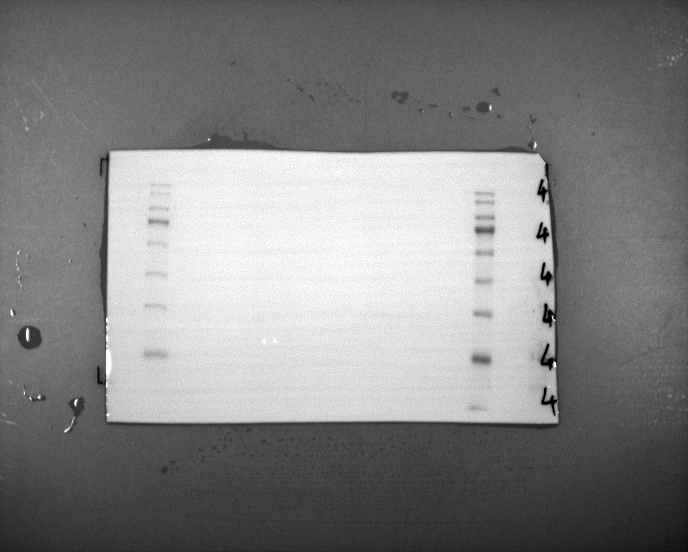

Supplement: Supplemental Information 10 [file peerj-12-16748-s010.zip › 4.tif]

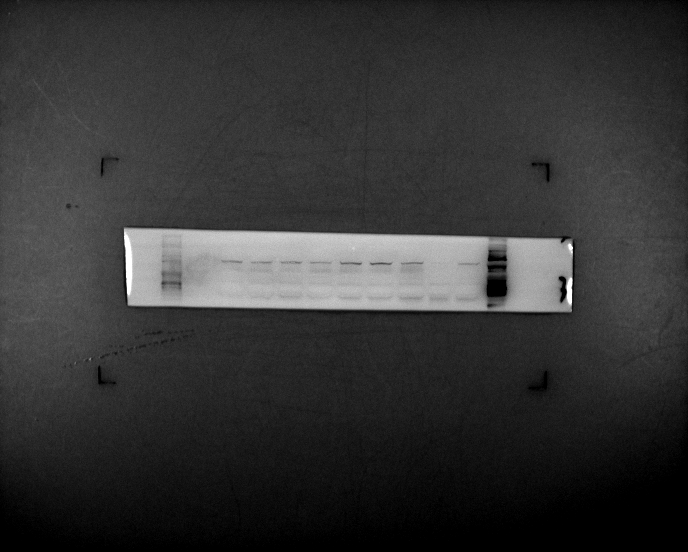

Supplement: Supplemental Information 10 [file peerj-12-16748-s010.zip › ACSL4-2.tif]

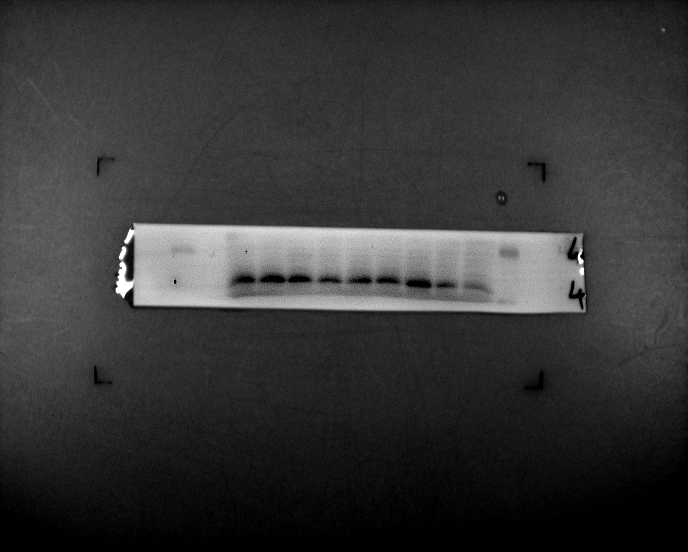

Supplement: Supplemental Information 10 [file peerj-12-16748-s010.zip › FTH4.tif]

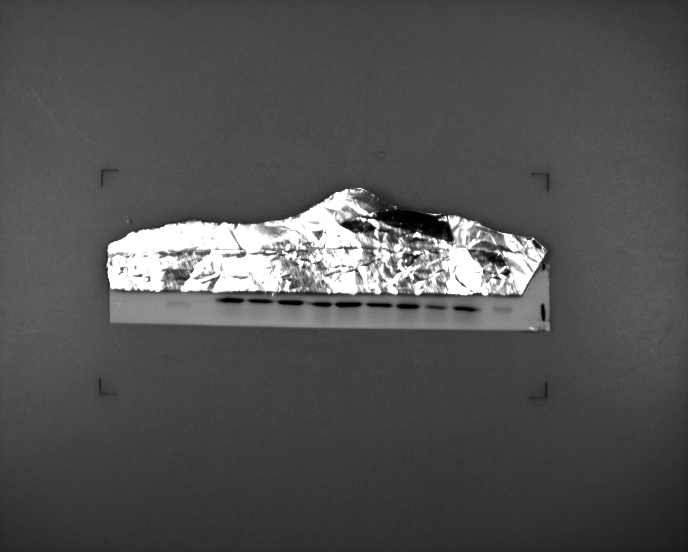

Supplement: Supplemental Information 10 [file peerj-12-16748-s010.zip › GAPDH1-2.tif]

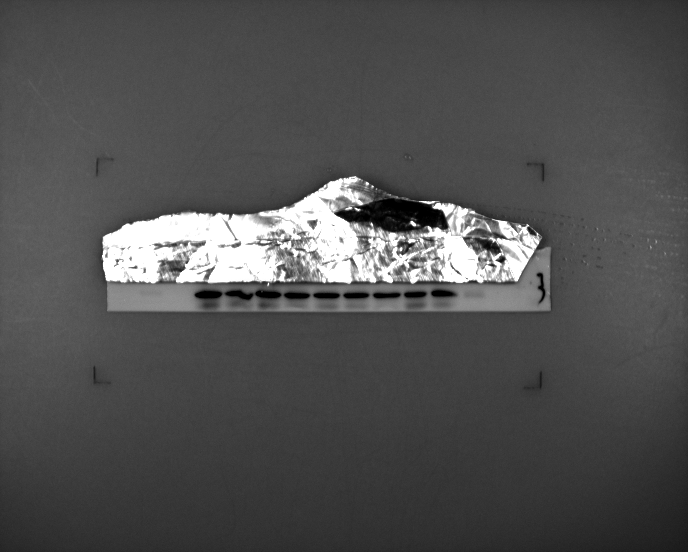

Supplement: Supplemental Information 10 [file peerj-12-16748-s010.zip › GAPDH3-2.tif]

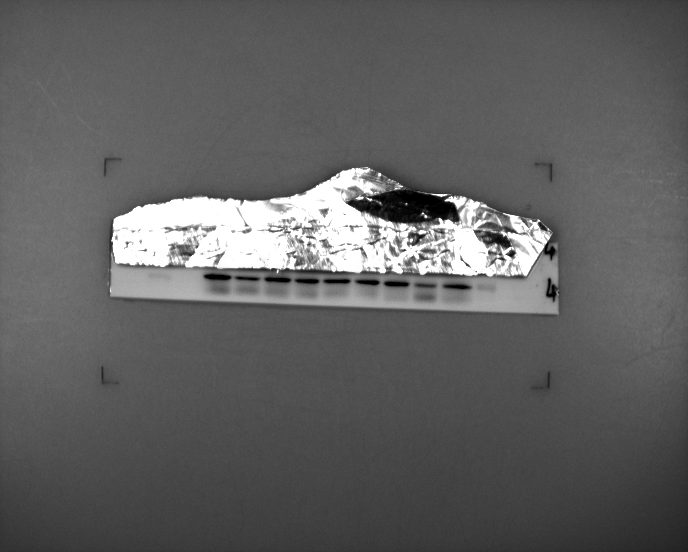

Supplement: Supplemental Information 10 [file peerj-12-16748-s010.zip › GAPDH4-2.tif]

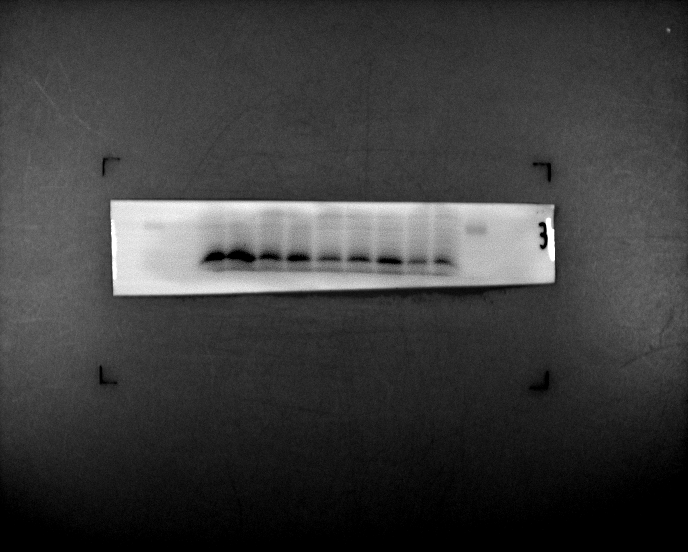

Supplement: Supplemental Information 10 [file peerj-12-16748-s010.zip › GPX4-2.tif]

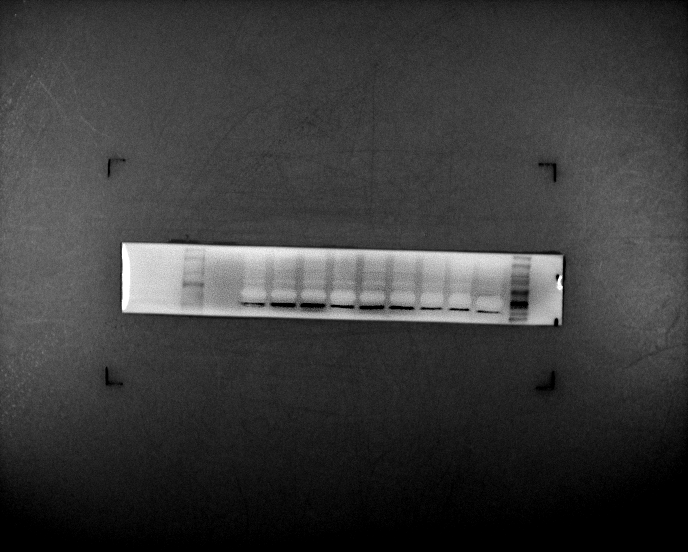

Supplement: Supplemental Information 10 [file peerj-12-16748-s010.zip › paD4-2.tif]

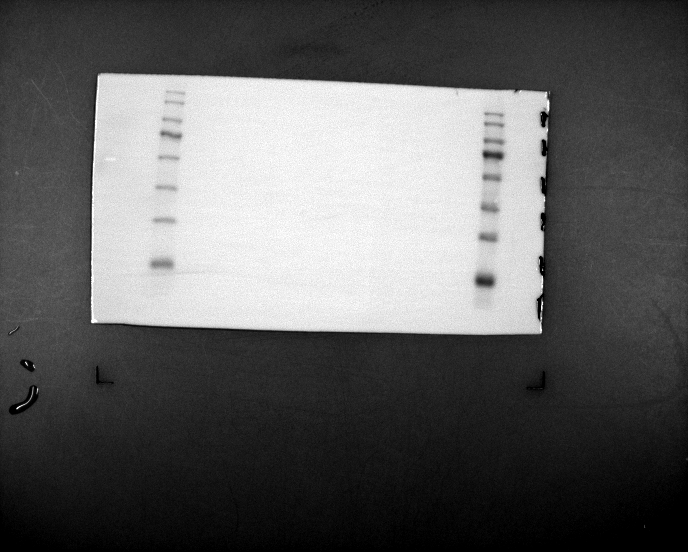

Supplement: Supplemental Information 11 [file peerj-12-16748-s011.zip › Figure3ú1⁄4WB/1.tif]

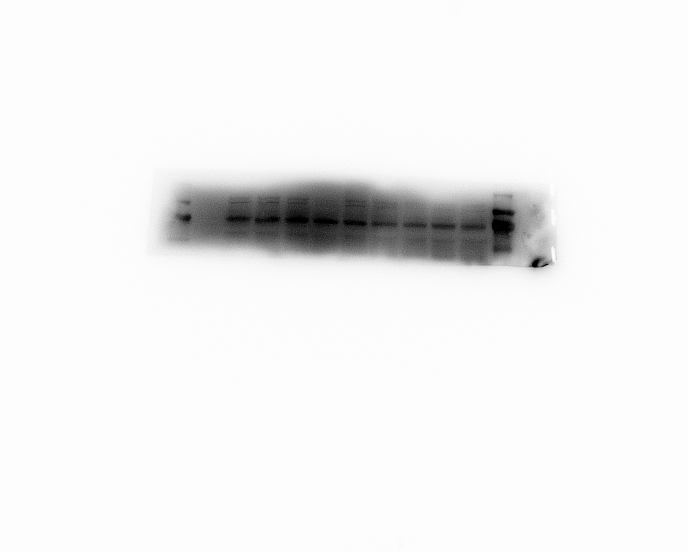

Supplement: Supplemental Information 11 [file peerj-12-16748-s011.zip › Figure3ú1⁄4WB/1-acsl4.tif]

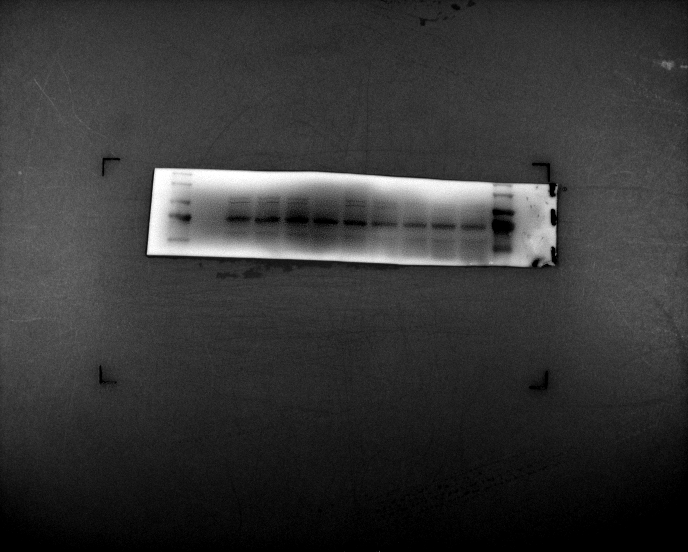

Supplement: Supplemental Information 11 [file peerj-12-16748-s011.zip › Figure3ú1⁄4WB/1-acsl4-2.tif]

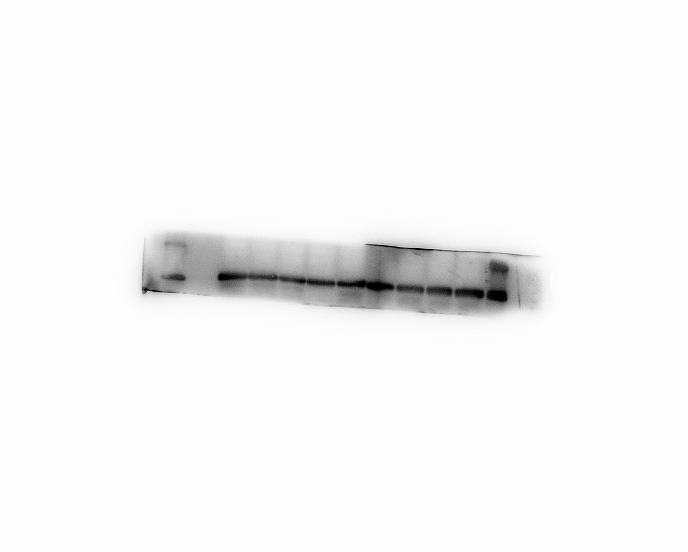

Supplement: Supplemental Information 11 [file peerj-12-16748-s011.zip › Figure3ú1⁄4WB/1-gapdh1.tif]

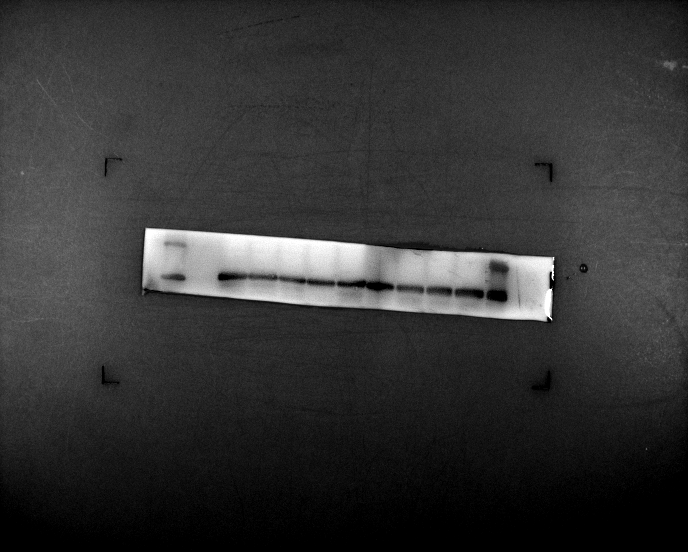

Supplement: Supplemental Information 11 [file peerj-12-16748-s011.zip › Figure3ú1⁄4WB/1-gapdh2.tif]

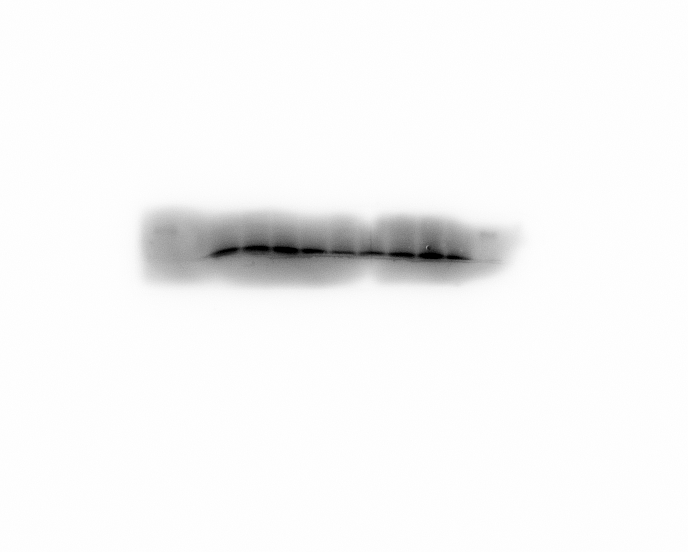

Supplement: Supplemental Information 11 [file peerj-12-16748-s011.zip › Figure3ú1⁄4WB/1-gpx4-5.tif]

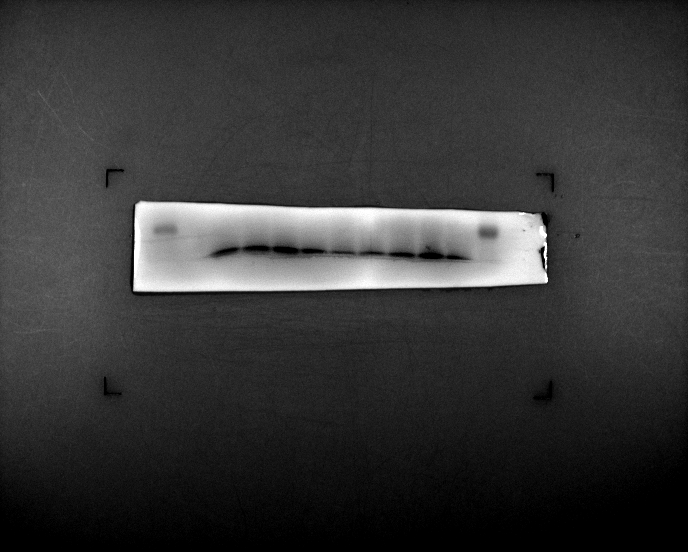

Supplement: Supplemental Information 11 [file peerj-12-16748-s011.zip › Figure3ú1⁄4WB/1-gpx4-6.tif]

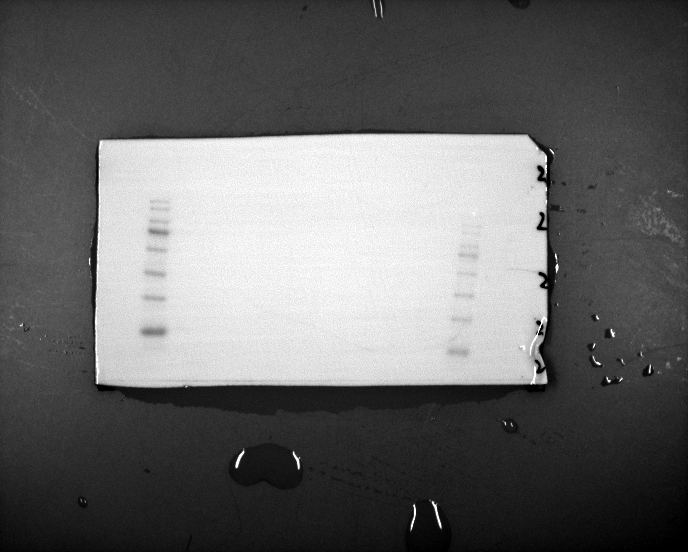

Supplement: Supplemental Information 11 [file peerj-12-16748-s011.zip › Figure3ú1⁄4WB/2.tif]

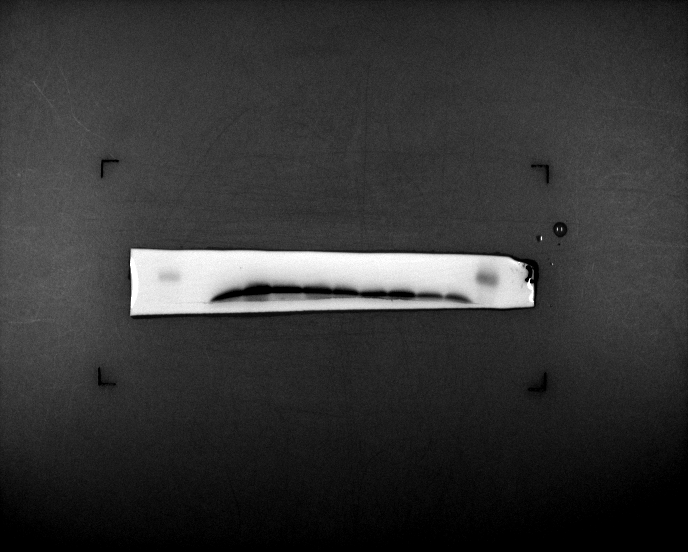

Supplement: Supplemental Information 11 [file peerj-12-16748-s011.zip › Figure3ú1⁄4WB/2-fth4.tif]

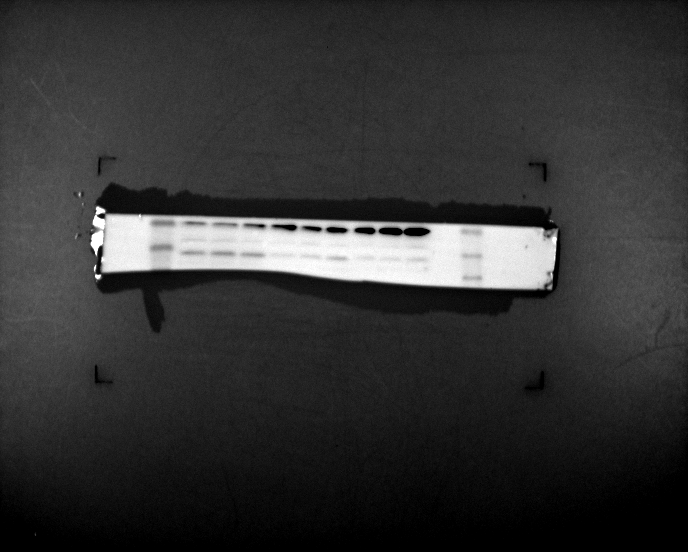

Supplement: Supplemental Information 11 [file peerj-12-16748-s011.zip › Figure3ú1⁄4WB/2-GAPDH.tif]

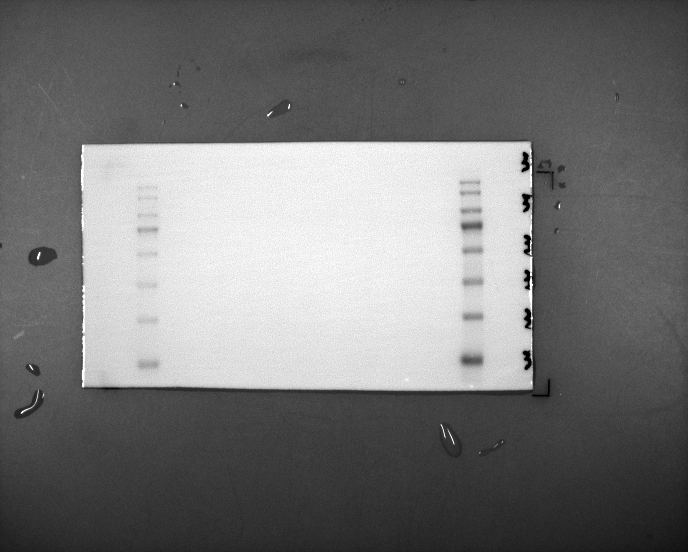

Supplement: Supplemental Information 11 [file peerj-12-16748-s011.zip › Figure3ú1⁄4WB/3.tif]

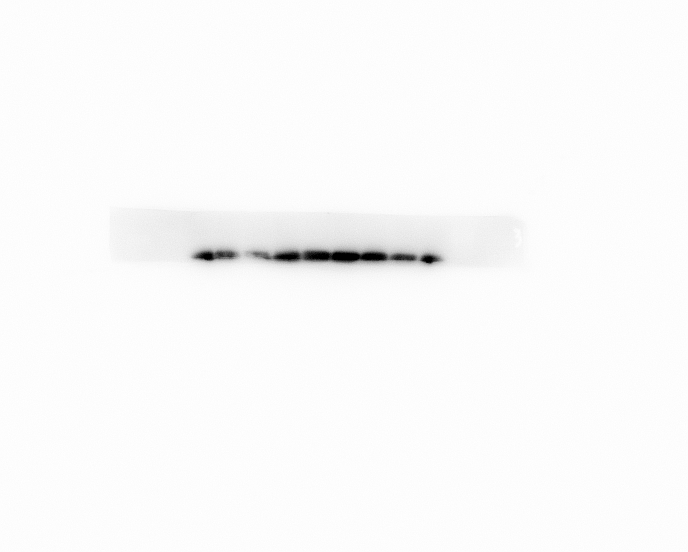

Supplement: Supplemental Information 11 [file peerj-12-16748-s011.zip › Figure3ú1⁄4WB/3-fth1.tif]

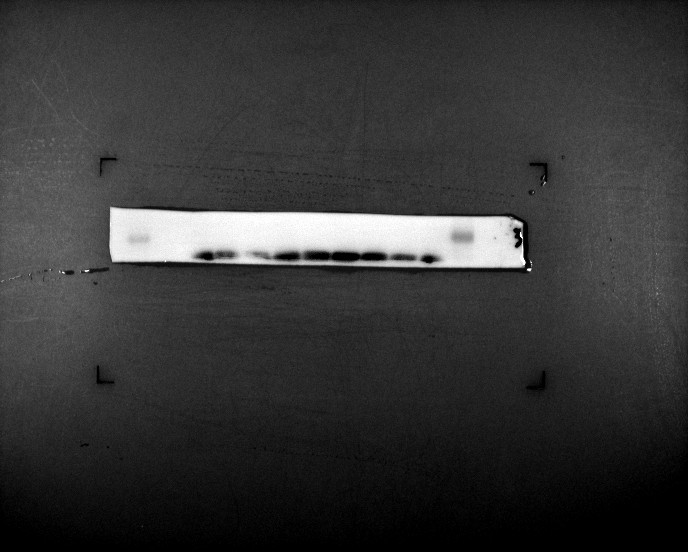

Supplement: Supplemental Information 11 [file peerj-12-16748-s011.zip › Figure3ú1⁄4WB/3-fth2.tif]

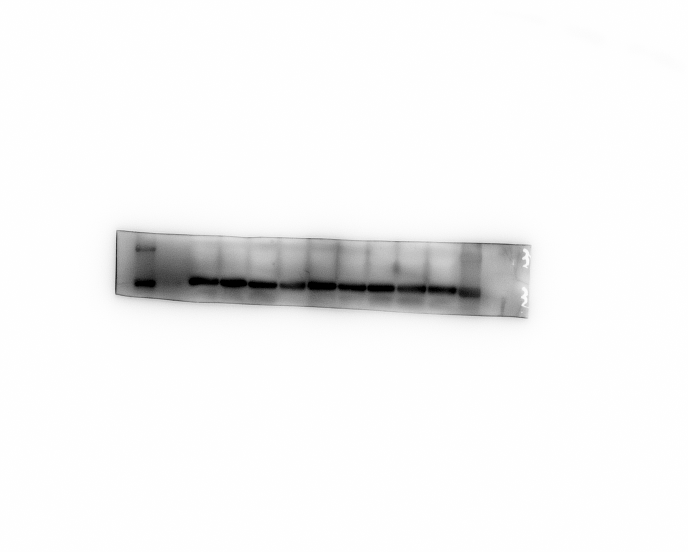

Supplement: Supplemental Information 11 [file peerj-12-16748-s011.zip › Figure3ú1⁄4WB/3-gapdh1.tif]

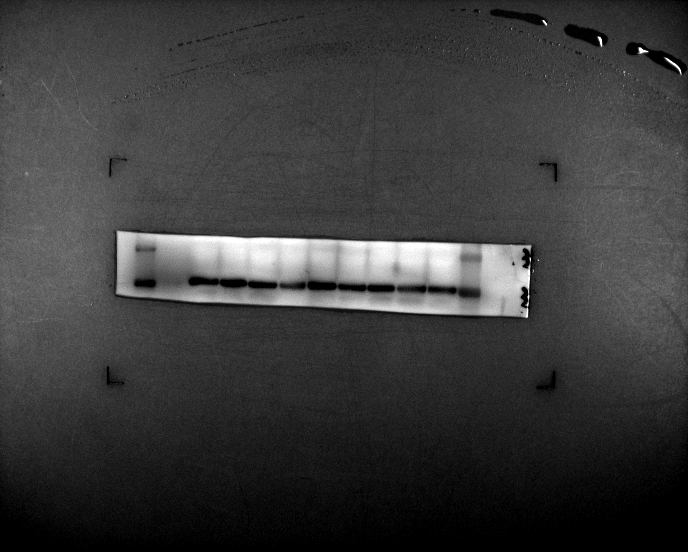

Supplement: Supplemental Information 11 [file peerj-12-16748-s011.zip › Figure3ú1⁄4WB/3-gapdh2.tif]

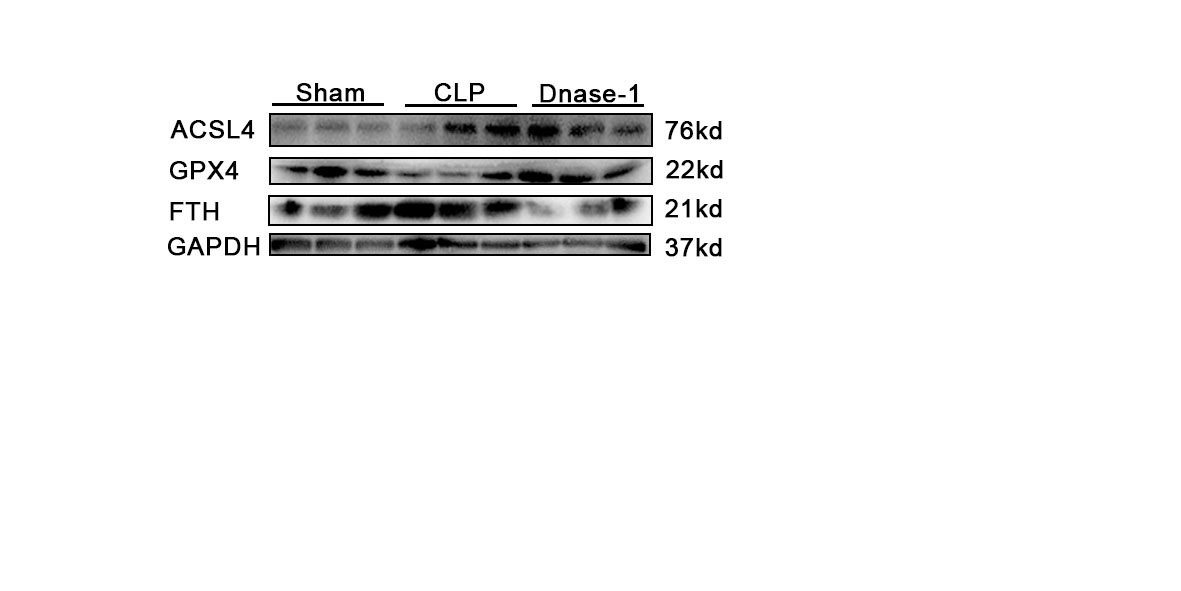

Supplement: Supplemental Information 11 [file peerj-12-16748-s011.zip › Figure3ú1⁄4WB/WB-3.jpg]

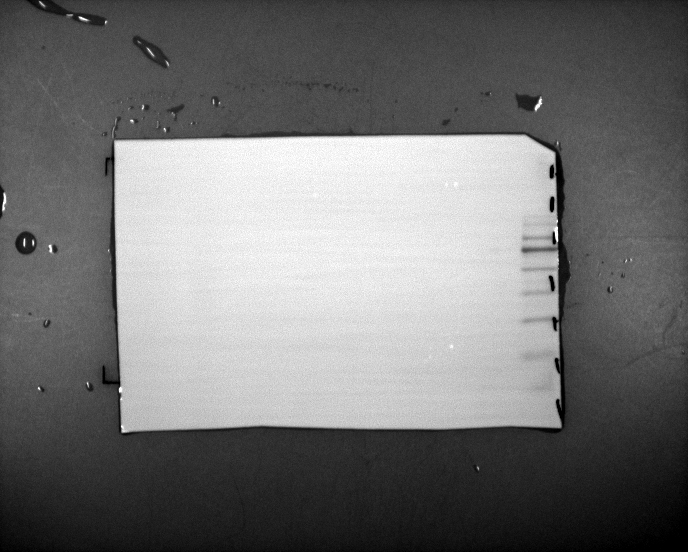

Supplement: Supplemental Information 12 [file peerj-12-16748-s012.zip › 1.tif]

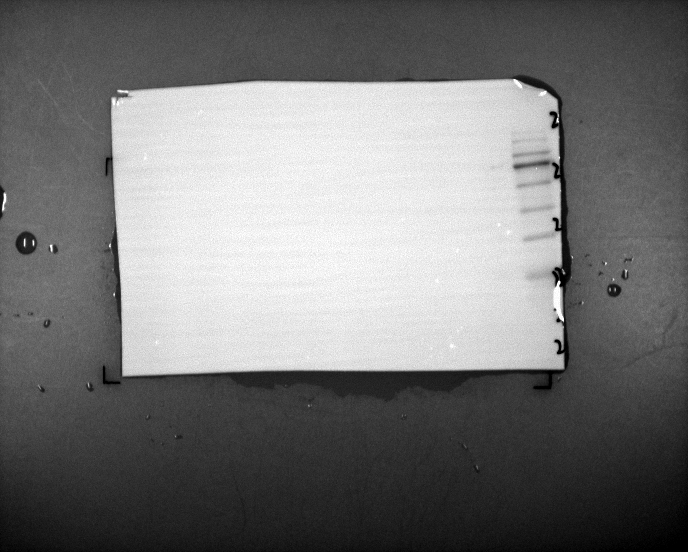

Supplement: Supplemental Information 12 [file peerj-12-16748-s012.zip › 2.tif]

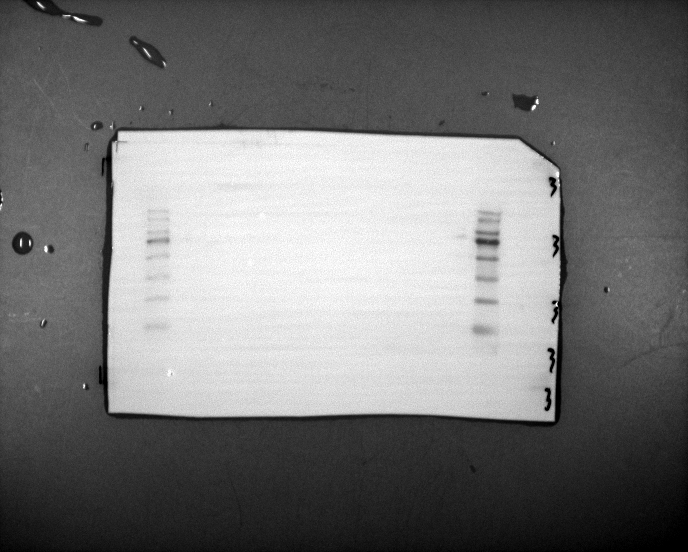

Supplement: Supplemental Information 12 [file peerj-12-16748-s012.zip › 3.tif]

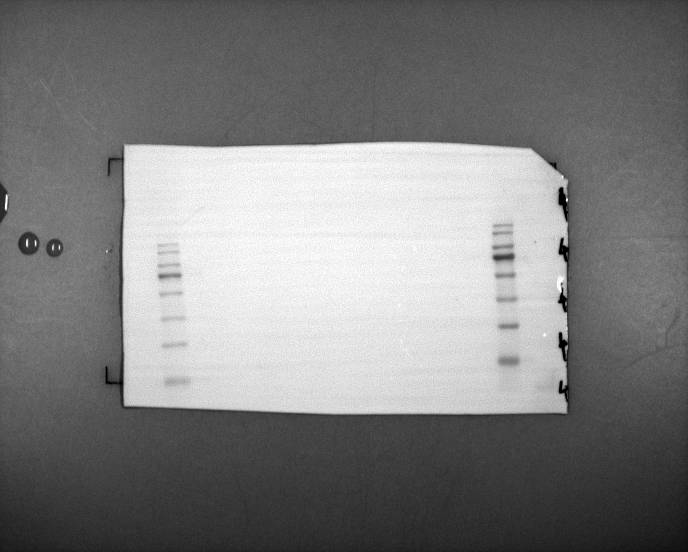

Supplement: Supplemental Information 12 [file peerj-12-16748-s012.zip › 4.tif]

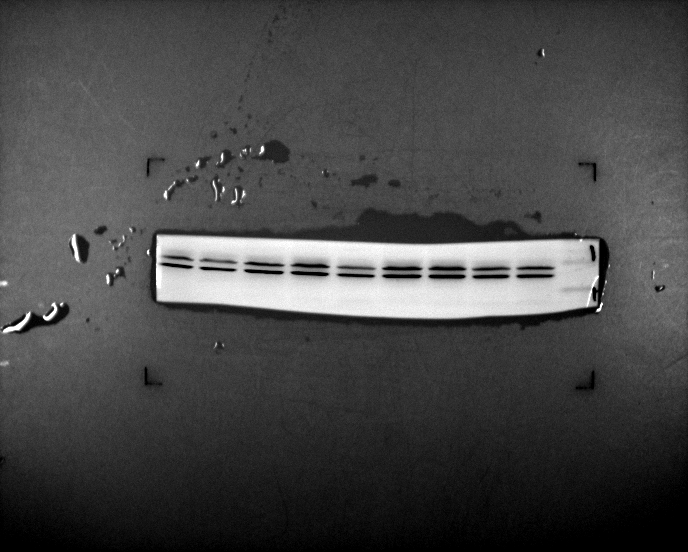

Supplement: Supplemental Information 12 [file peerj-12-16748-s012.zip › ERK4.tif]

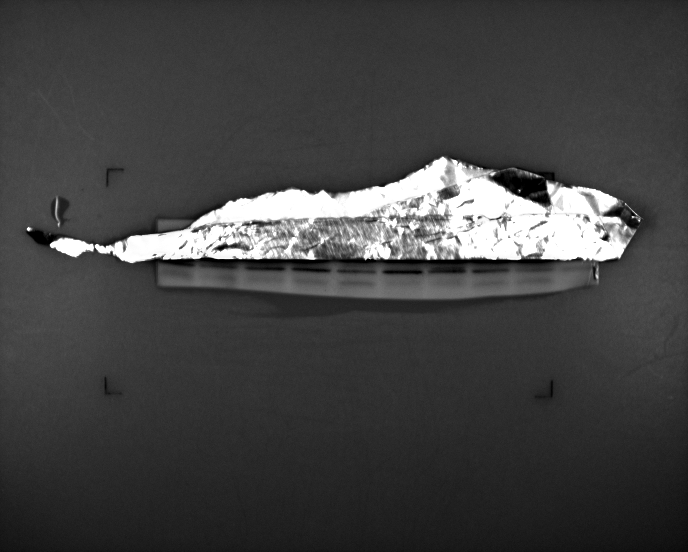

Supplement: Supplemental Information 12 [file peerj-12-16748-s012.zip › gapdh1-2.tif]

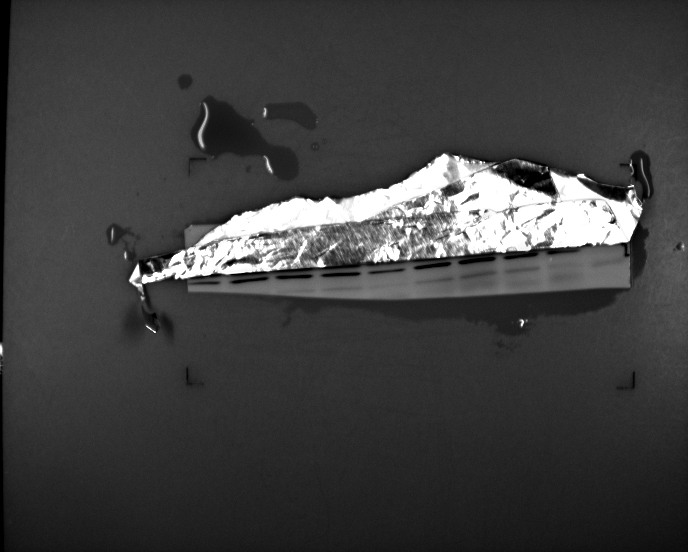

Supplement: Supplemental Information 12 [file peerj-12-16748-s012.zip › gapdh2-2.tif]

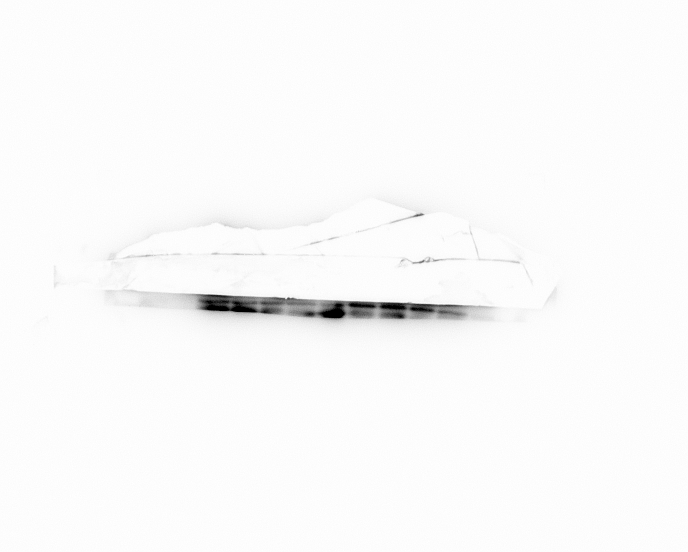

Supplement: Supplemental Information 12 [file peerj-12-16748-s012.zip › gapdh3-1.tif]

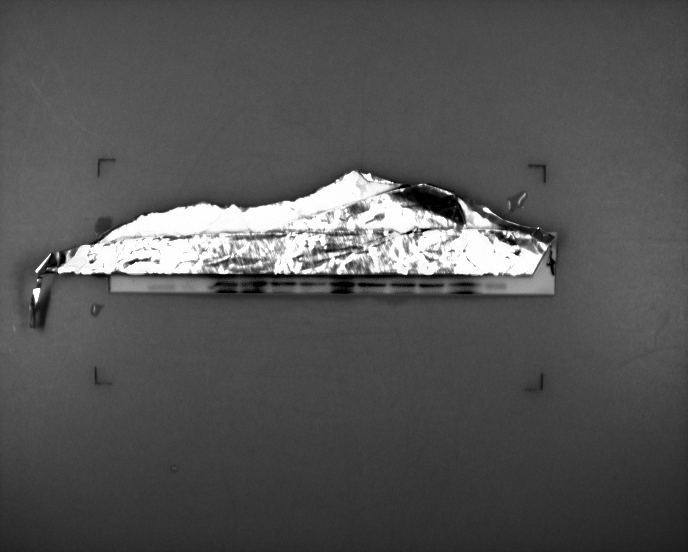

Supplement: Supplemental Information 12 [file peerj-12-16748-s012.zip › gapdh4-2.tif]

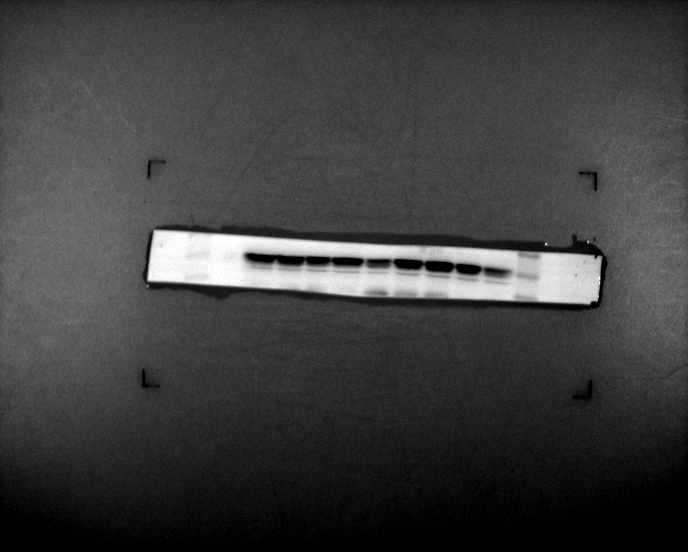

Supplement: Supplemental Information 12 [file peerj-12-16748-s012.zip › MEK4.tif]

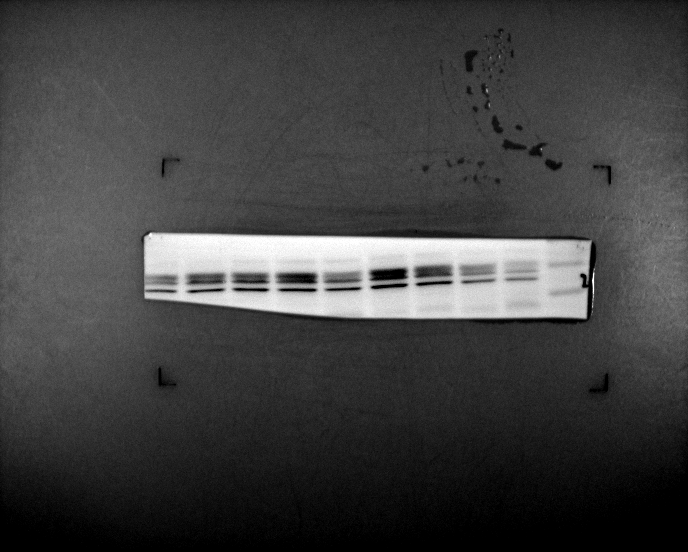

Supplement: Supplemental Information 12 [file peerj-12-16748-s012.zip › PERK2.tif]

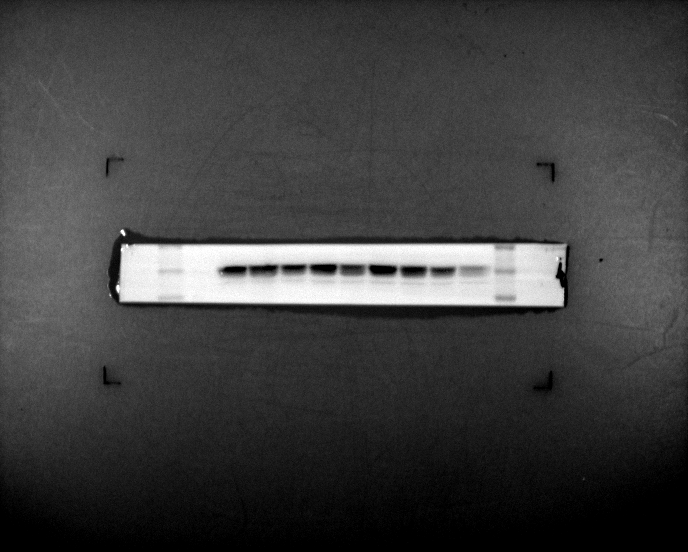

Supplement: Supplemental Information 12 [file peerj-12-16748-s012.zip › PMEK4.tif]

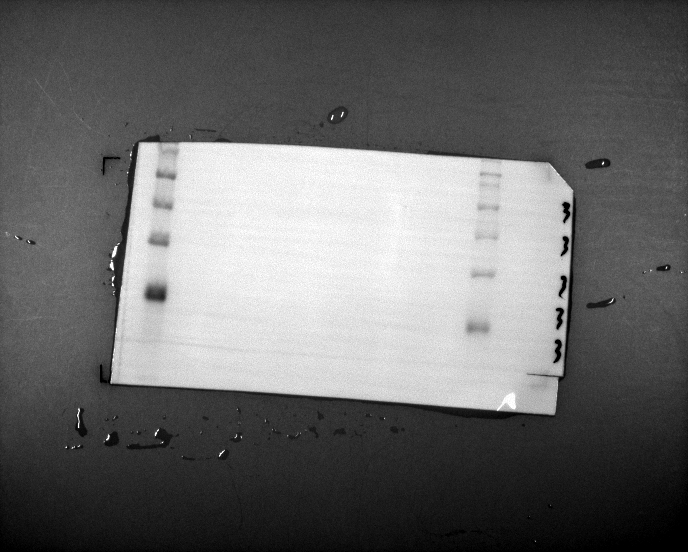

Supplement: Supplemental Information 13 [file peerj-12-16748-s013.zip › Figure 5ú1⁄4WB/3-M1.tif]

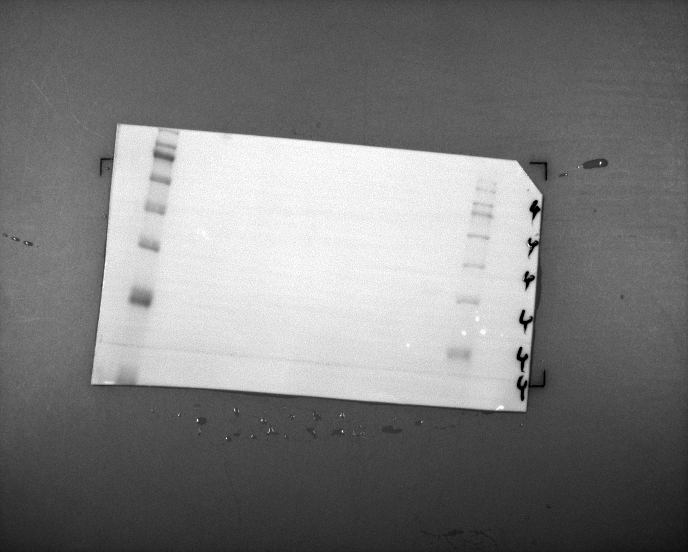

Supplement: Supplemental Information 13 [file peerj-12-16748-s013.zip › Figure 5ú1⁄4WB/4-M,1.tif]

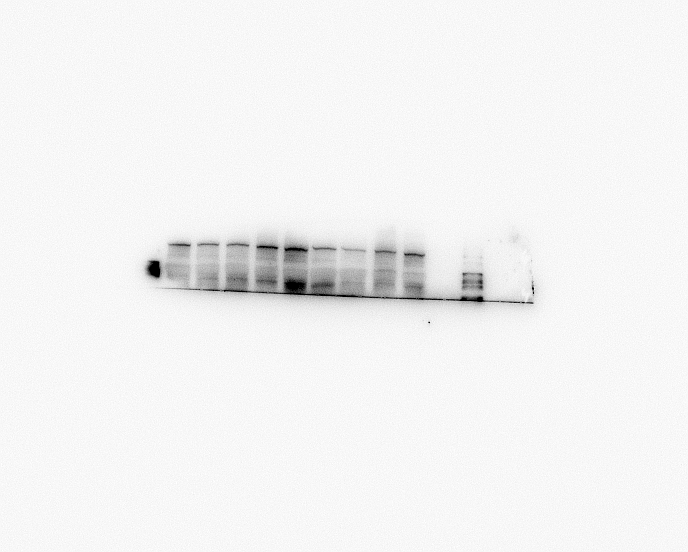

Supplement: Supplemental Information 13 [file peerj-12-16748-s013.zip › Figure 5ú1⁄4WB/acsl4-1.tif]

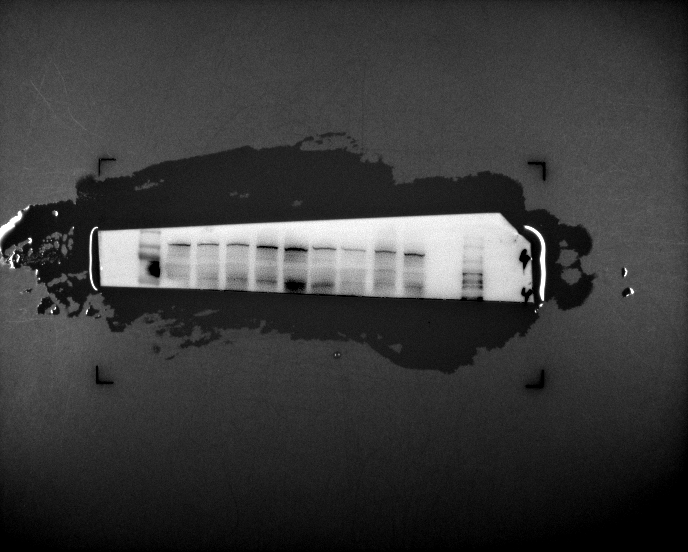

Supplement: Supplemental Information 13 [file peerj-12-16748-s013.zip › Figure 5ú1⁄4WB/acsl4-2.tif]

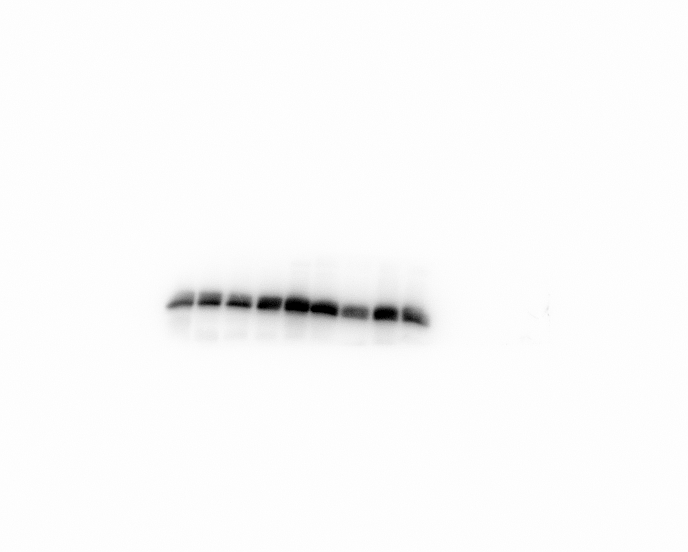

Supplement: Supplemental Information 13 [file peerj-12-16748-s013.zip › Figure 5ú1⁄4WB/fth-1.tif]

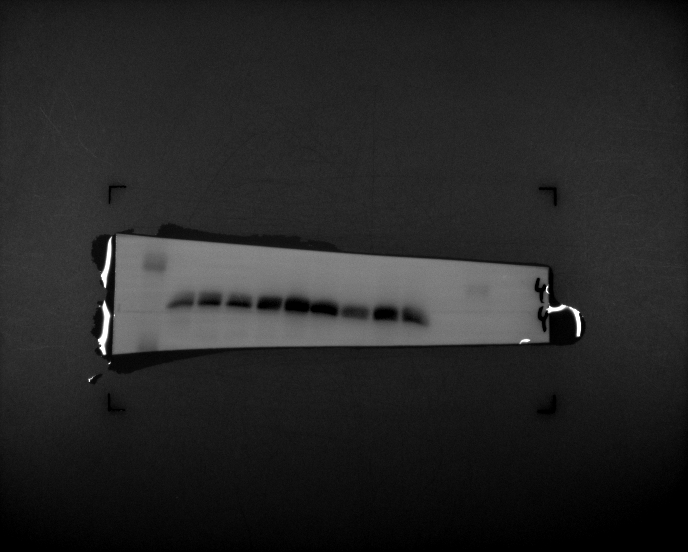

Supplement: Supplemental Information 13 [file peerj-12-16748-s013.zip › Figure 5ú1⁄4WB/fth-2.tif]

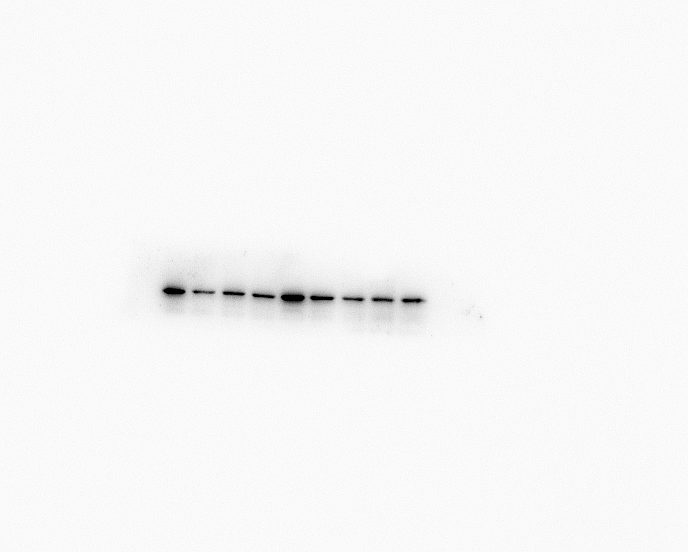

Supplement: Supplemental Information 13 [file peerj-12-16748-s013.zip › Figure 5ú1⁄4WB/gapdh3-1.tif]

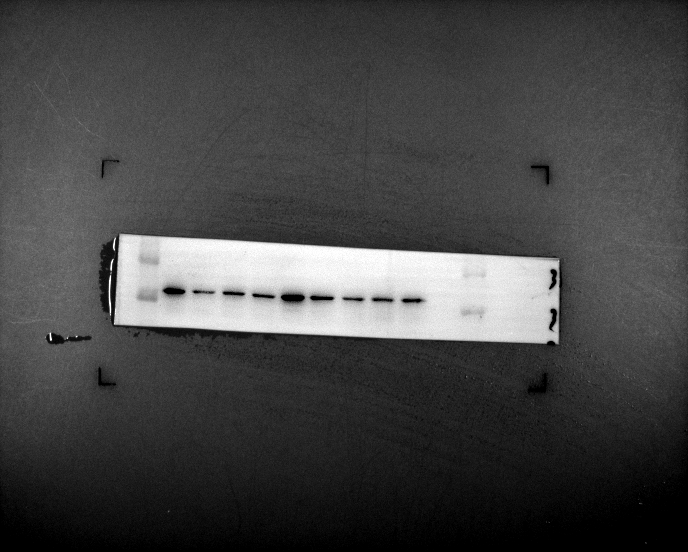

Supplement: Supplemental Information 13 [file peerj-12-16748-s013.zip › Figure 5ú1⁄4WB/gapdh3-2.tif]

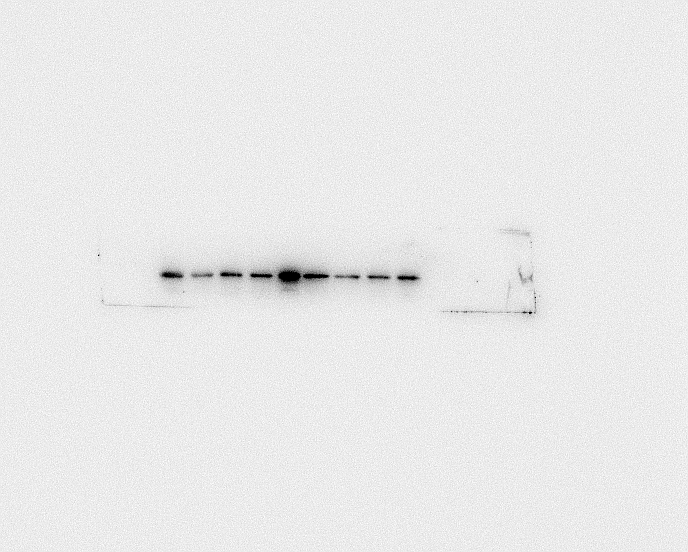

Supplement: Supplemental Information 13 [file peerj-12-16748-s013.zip › Figure 5ú1⁄4WB/gapdh4-1.tif]

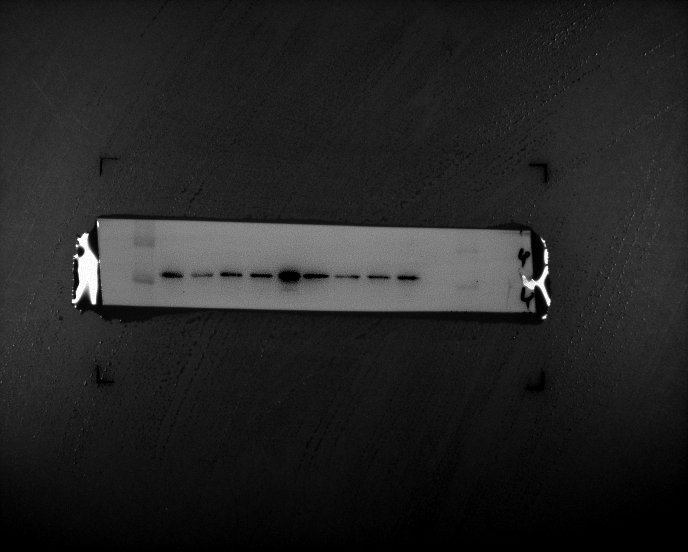

Supplement: Supplemental Information 13 [file peerj-12-16748-s013.zip › Figure 5ú1⁄4WB/gapdh4-2.tif]

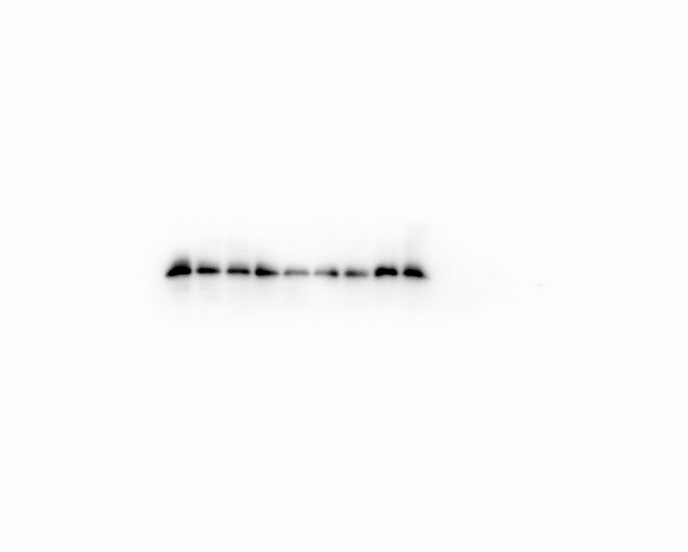

Supplement: Supplemental Information 13 [file peerj-12-16748-s013.zip › Figure 5ú1⁄4WB/gpx4-1.tif]

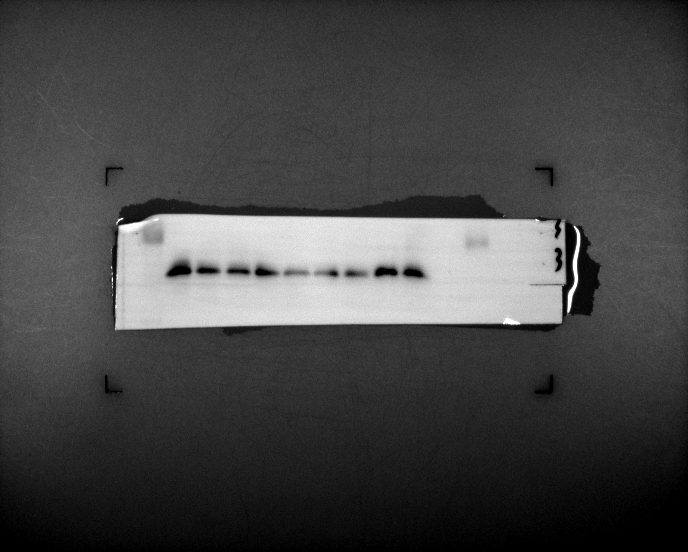

Supplement: Supplemental Information 13 [file peerj-12-16748-s013.zip › Figure 5ú1⁄4WB/gpx4-2.tif]

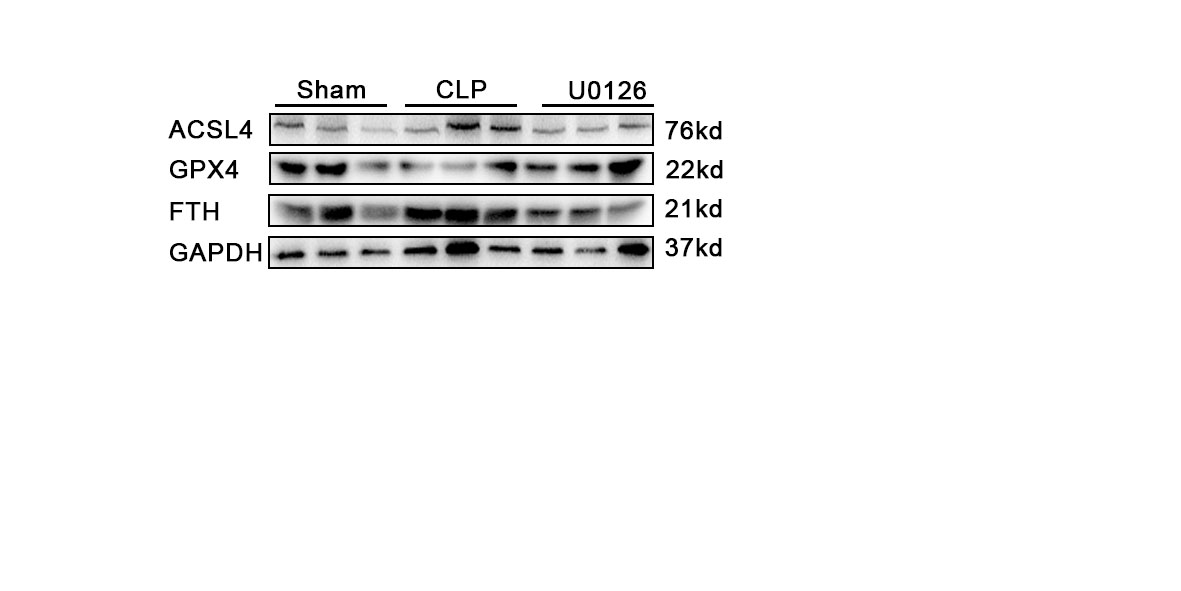

Supplement: Supplemental Information 13 [file peerj-12-16748-s013.zip › Figure 5ú1⁄4WB/WB-5F.jpg]

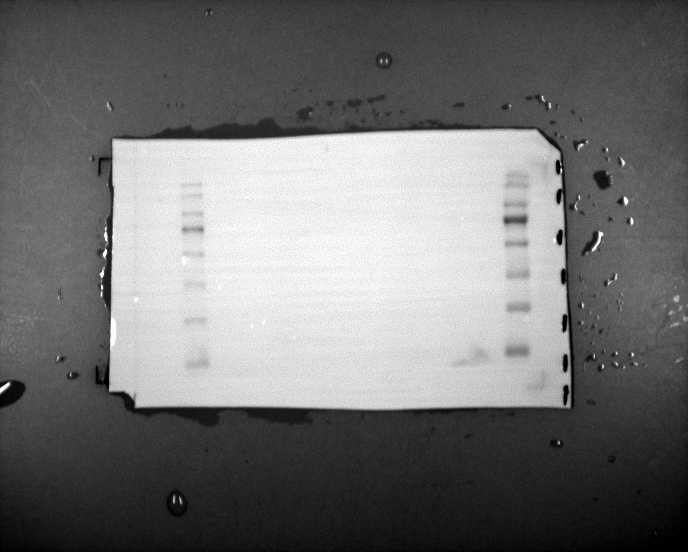

Supplement: Supplemental Information 14 [file peerj-12-16748-s014.zip › 1.tif]

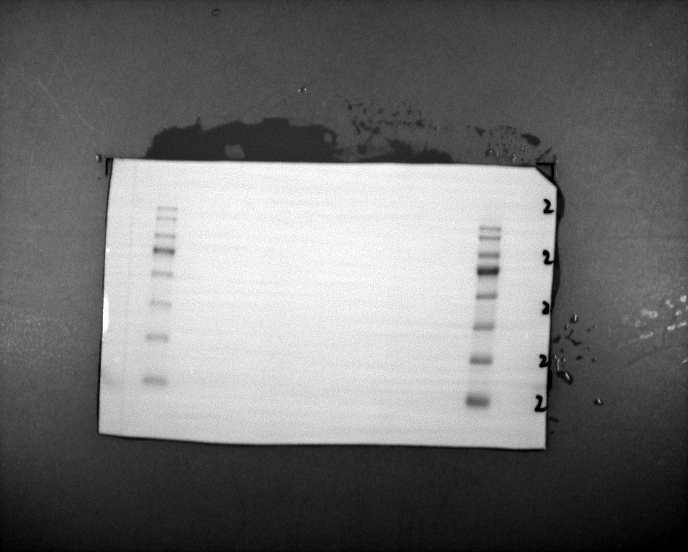

Supplement: Supplemental Information 14 [file peerj-12-16748-s014.zip › 2.tif]

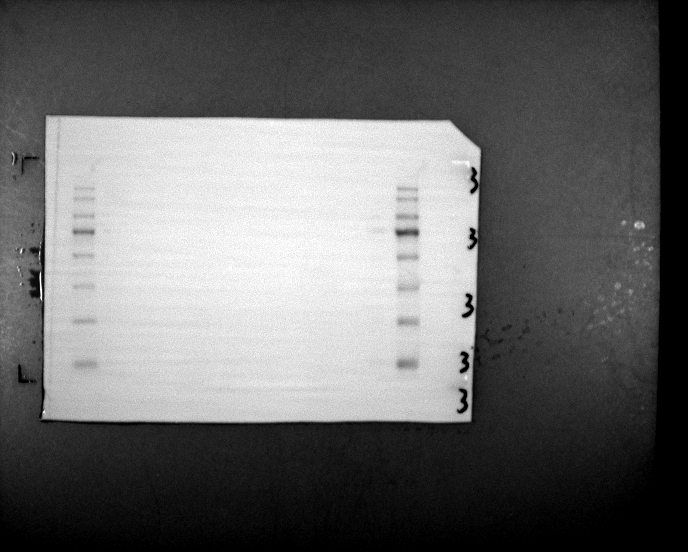

Supplement: Supplemental Information 14 [file peerj-12-16748-s014.zip › 3.tif]

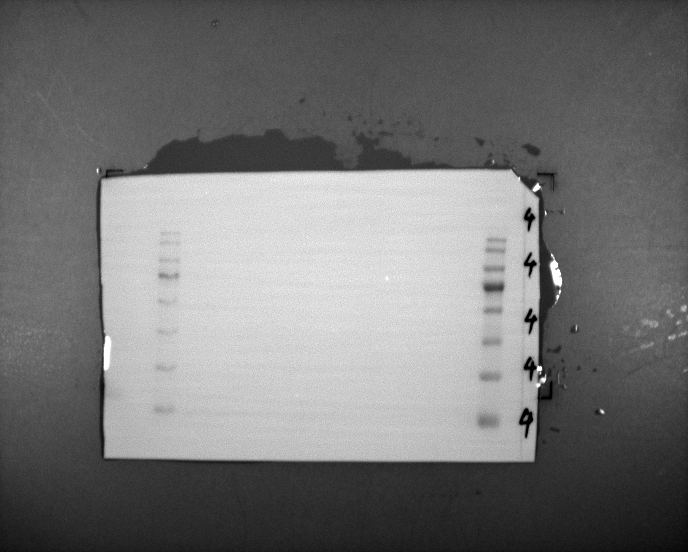

Supplement: Supplemental Information 14 [file peerj-12-16748-s014.zip › 4.tif]

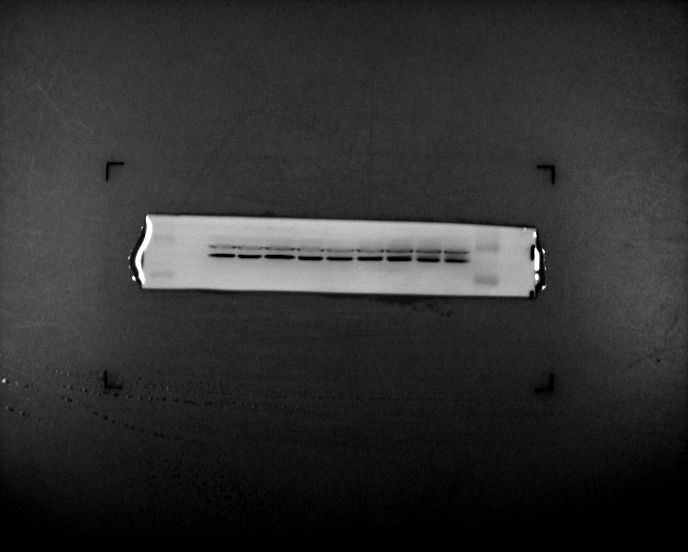

Supplement: Supplemental Information 14 [file peerj-12-16748-s014.zip › erk2.tif]

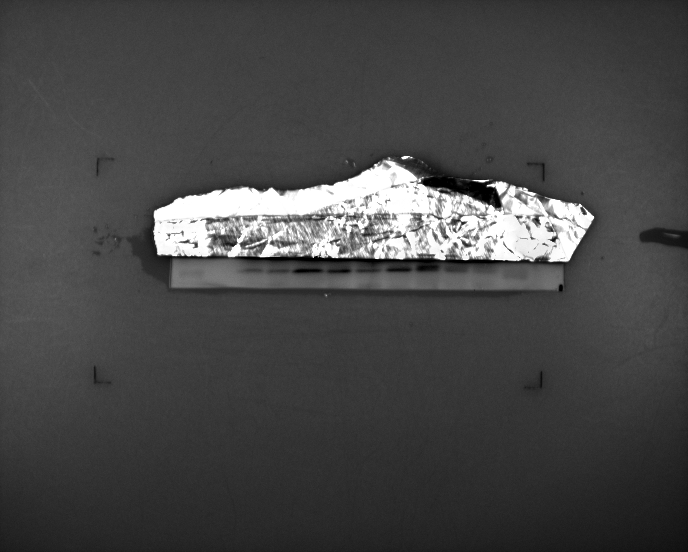

Supplement: Supplemental Information 14 [file peerj-12-16748-s014.zip › gapdh1-2.tif]

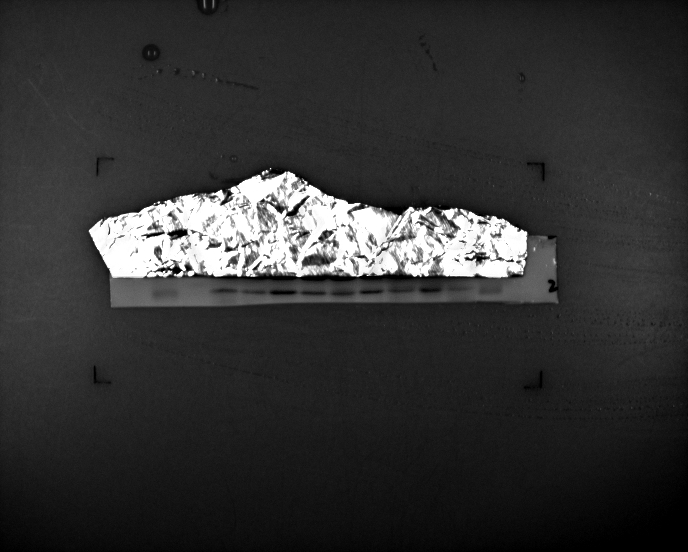

Supplement: Supplemental Information 14 [file peerj-12-16748-s014.zip › gapdh2-2.tif]

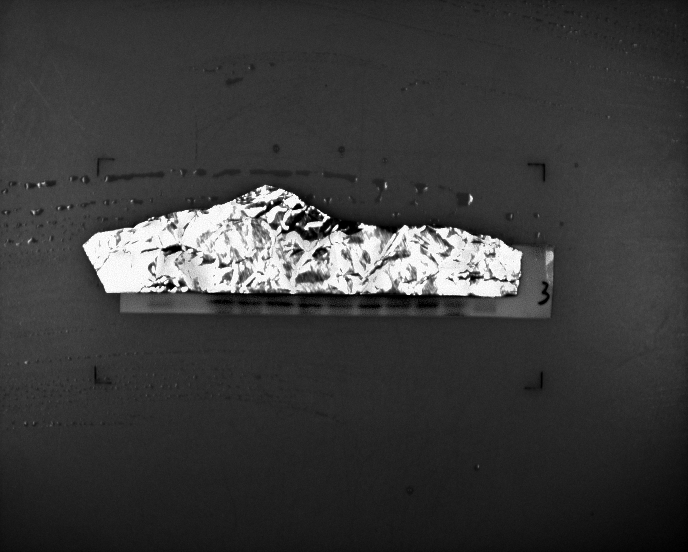

Supplement: Supplemental Information 14 [file peerj-12-16748-s014.zip › gapdh3-2.tif]

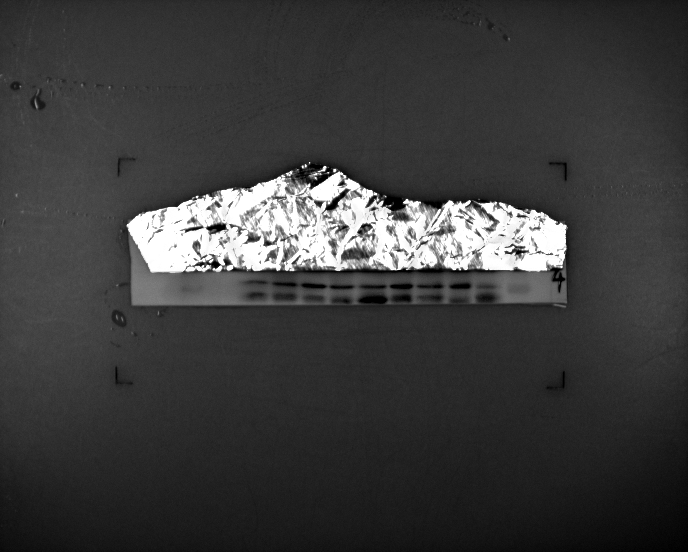

Supplement: Supplemental Information 14 [file peerj-12-16748-s014.zip › gapdh4-2.tif]

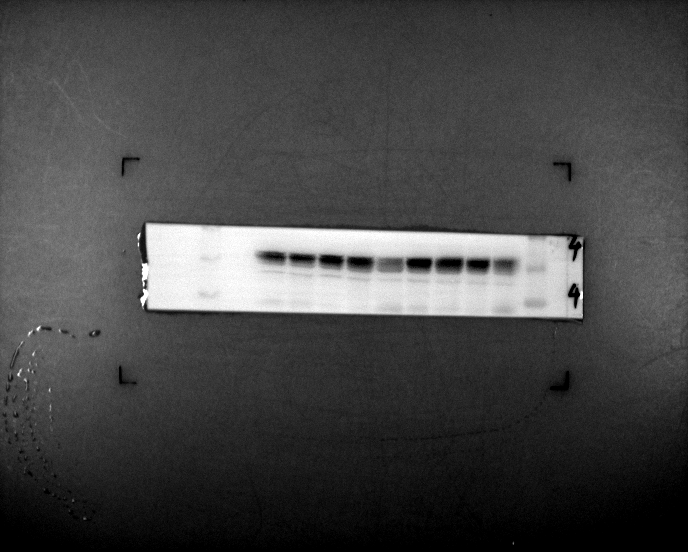

Supplement: Supplemental Information 14 [file peerj-12-16748-s014.zip › mek2.tif]

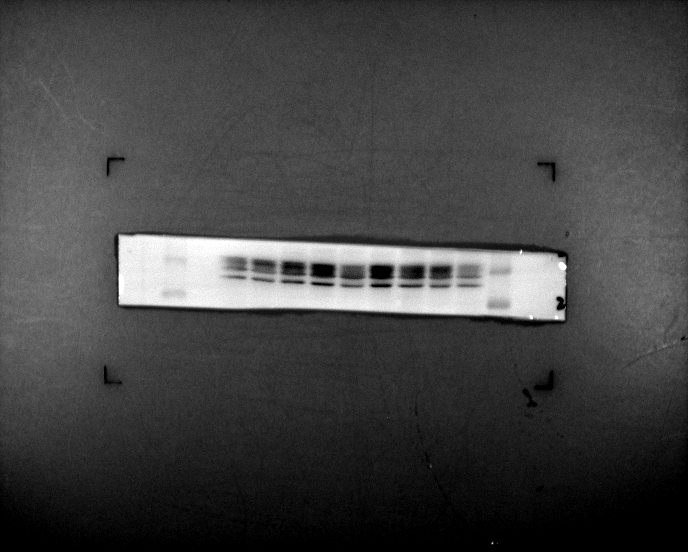

Supplement: Supplemental Information 14 [file peerj-12-16748-s014.zip › perk4.tif]

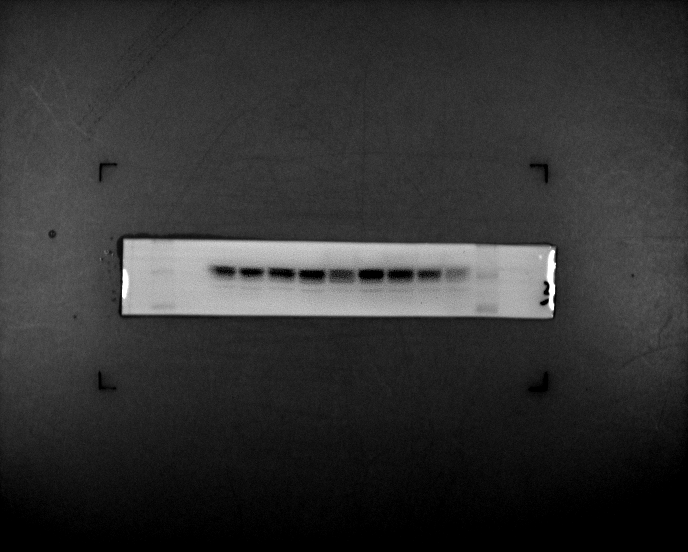

Supplement: Supplemental Information 14 [file peerj-12-16748-s014.zip › pmek2.tif]

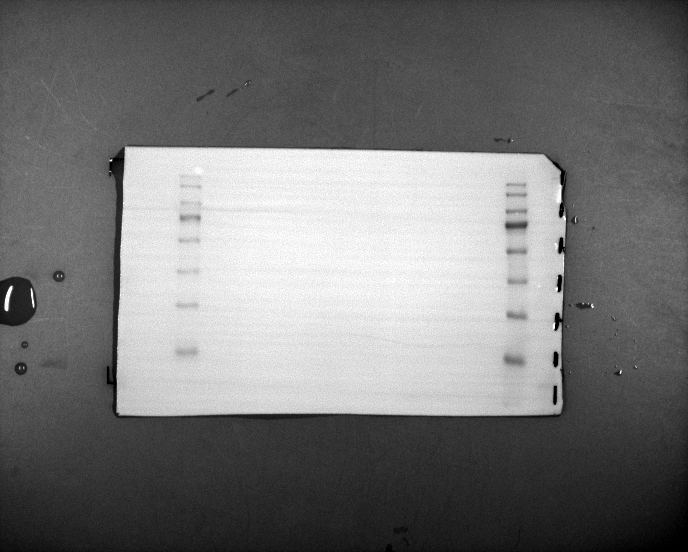

Supplement: Supplemental Information 15 [file peerj-12-16748-s015.zip › 1.tif]

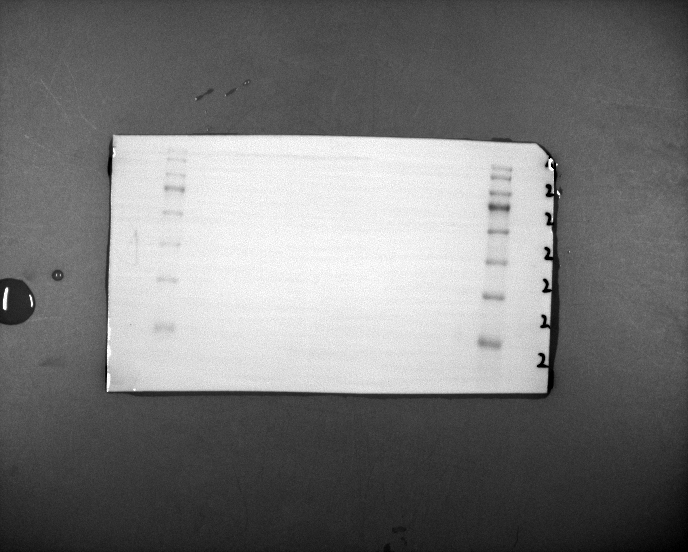

Supplement: Supplemental Information 15 [file peerj-12-16748-s015.zip › 2.tif]

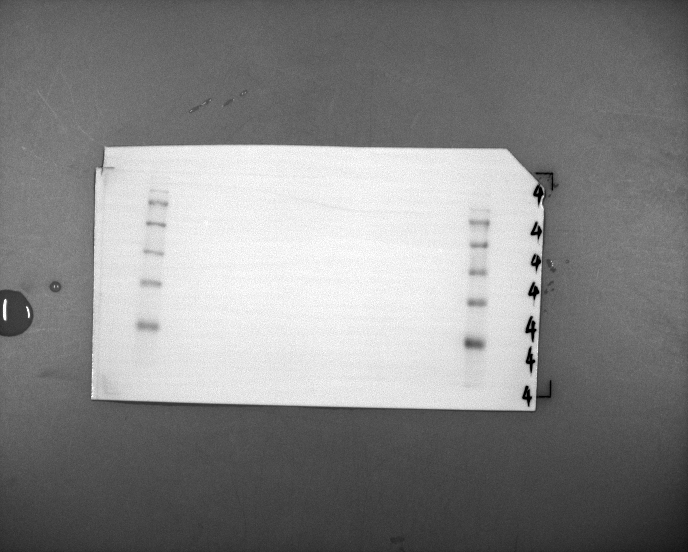

Supplement: Supplemental Information 15 [file peerj-12-16748-s015.zip › 4.tif]

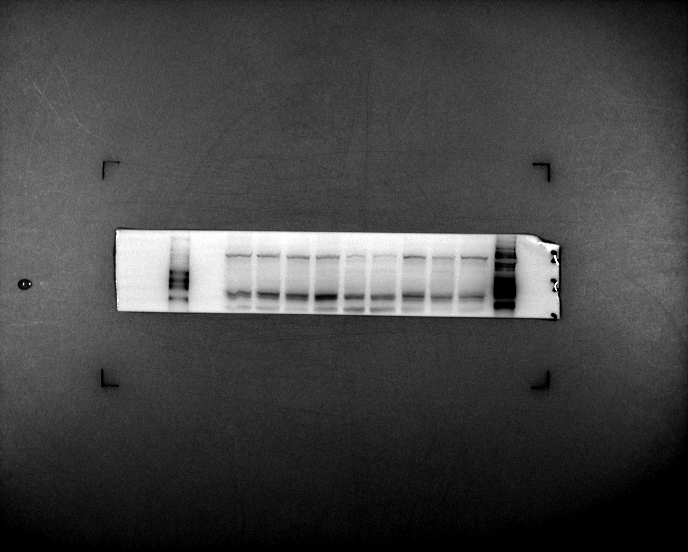

Supplement: Supplemental Information 15 [file peerj-12-16748-s015.zip › ACSL4-21.tif]

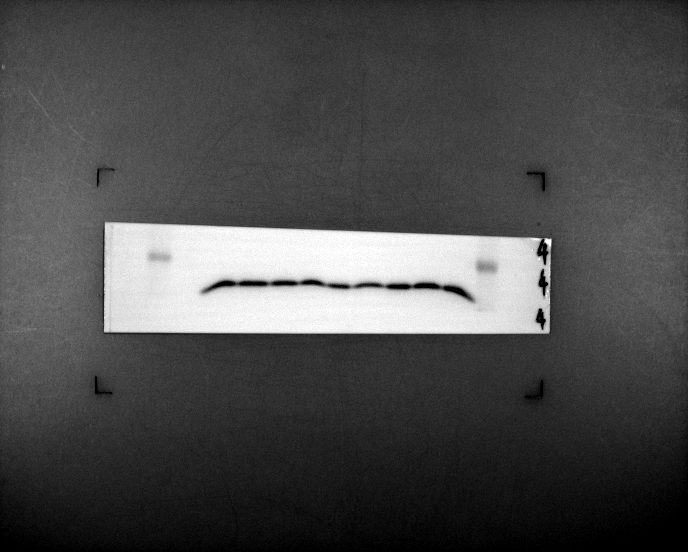

Supplement: Supplemental Information 15 [file peerj-12-16748-s015.zip › FTH-41.tif]

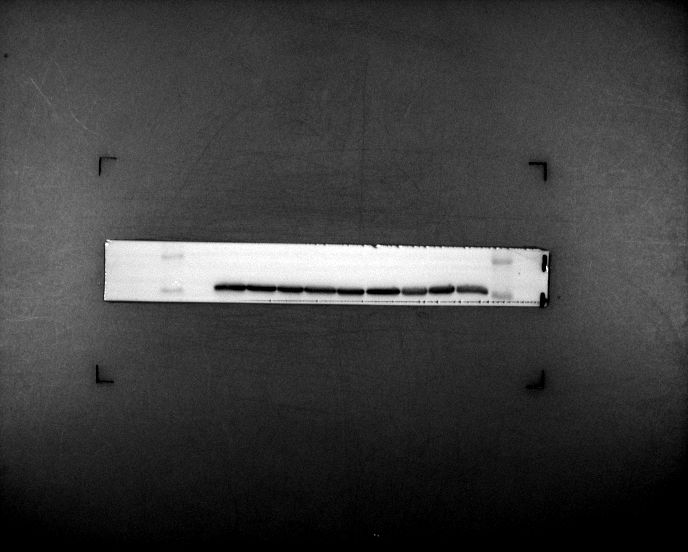

Supplement: Supplemental Information 15 [file peerj-12-16748-s015.zip › GAPDH-11.tif]

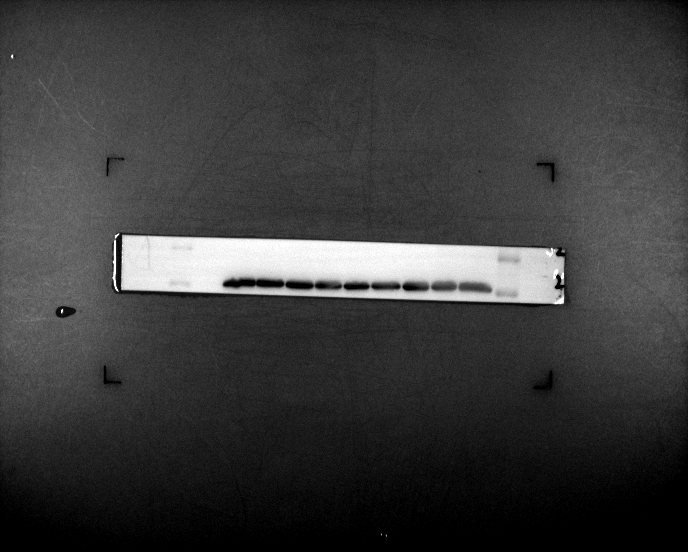

Supplement: Supplemental Information 15 [file peerj-12-16748-s015.zip › GAPDH-21.tif]

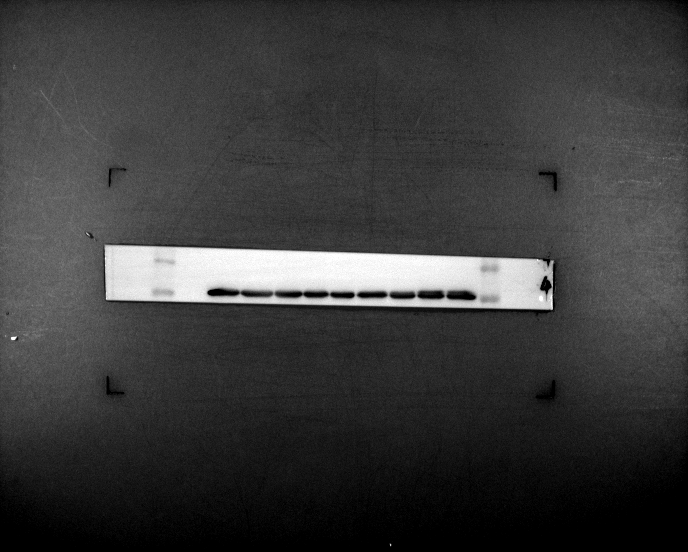

Supplement: Supplemental Information 15 [file peerj-12-16748-s015.zip › GAPDH-41.tif]

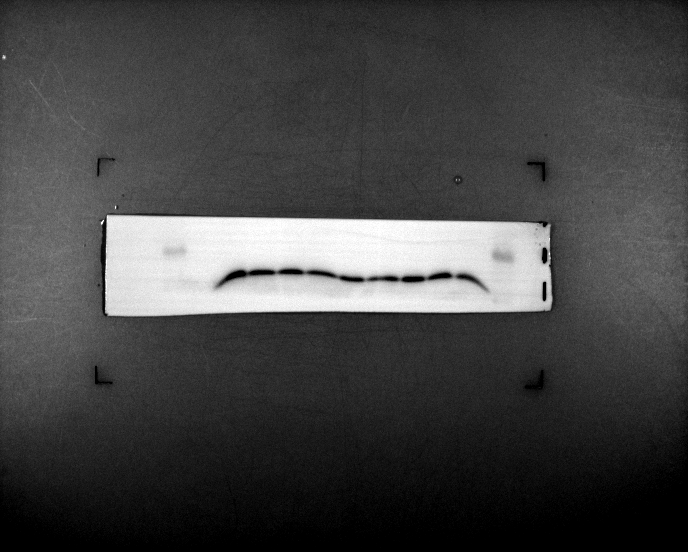

Supplement: Supplemental Information 15 [file peerj-12-16748-s015.zip › GPX4-11.tif]

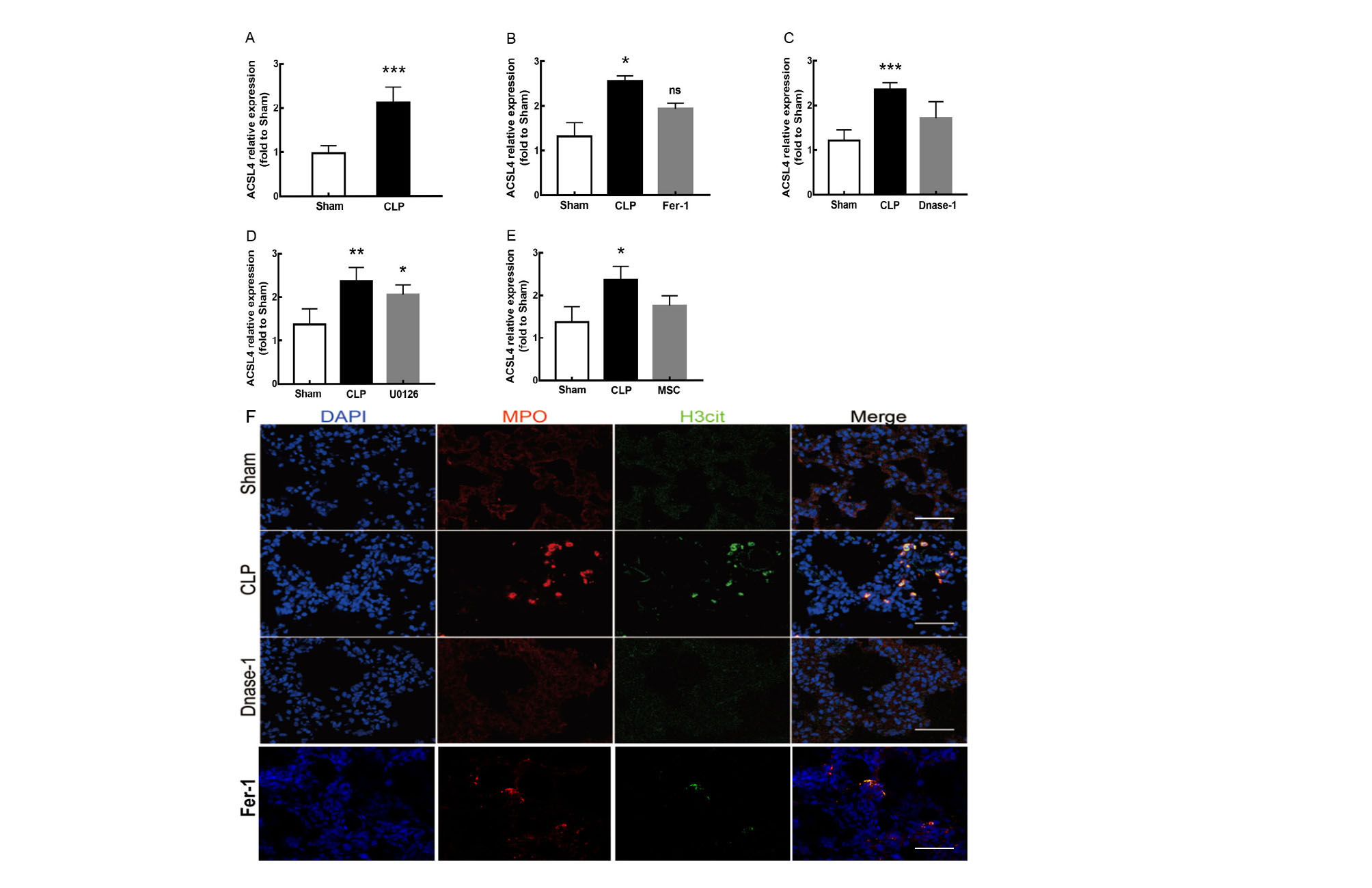

Supplement: Supplemental Information 16 — (A) Grayscale statistic of immunohistochemistry picture in figure 1. (*compared with Sham group, ***, p＜0.001) (B) Grayscale statistic of immunohistochemistry picture in figure 2. (*, p＜0.05; ns, no statistical significance) (C) Grayscale statistic of immunohistochemistry picture in figure 3. (***, p＜0.001) (D) Grayscale statistic of immunohistochemistry picture in figure 5 (*, p＜0.05; **, p < 0.01) (E) Grayscale statistic of immunohistochemistry picture in figure 7 (*, p＜0.05) (F) Nets formation in Sham, CLP, Dnase-1, Fer-1 group. [file peerj-12-16748-s016.jpg]

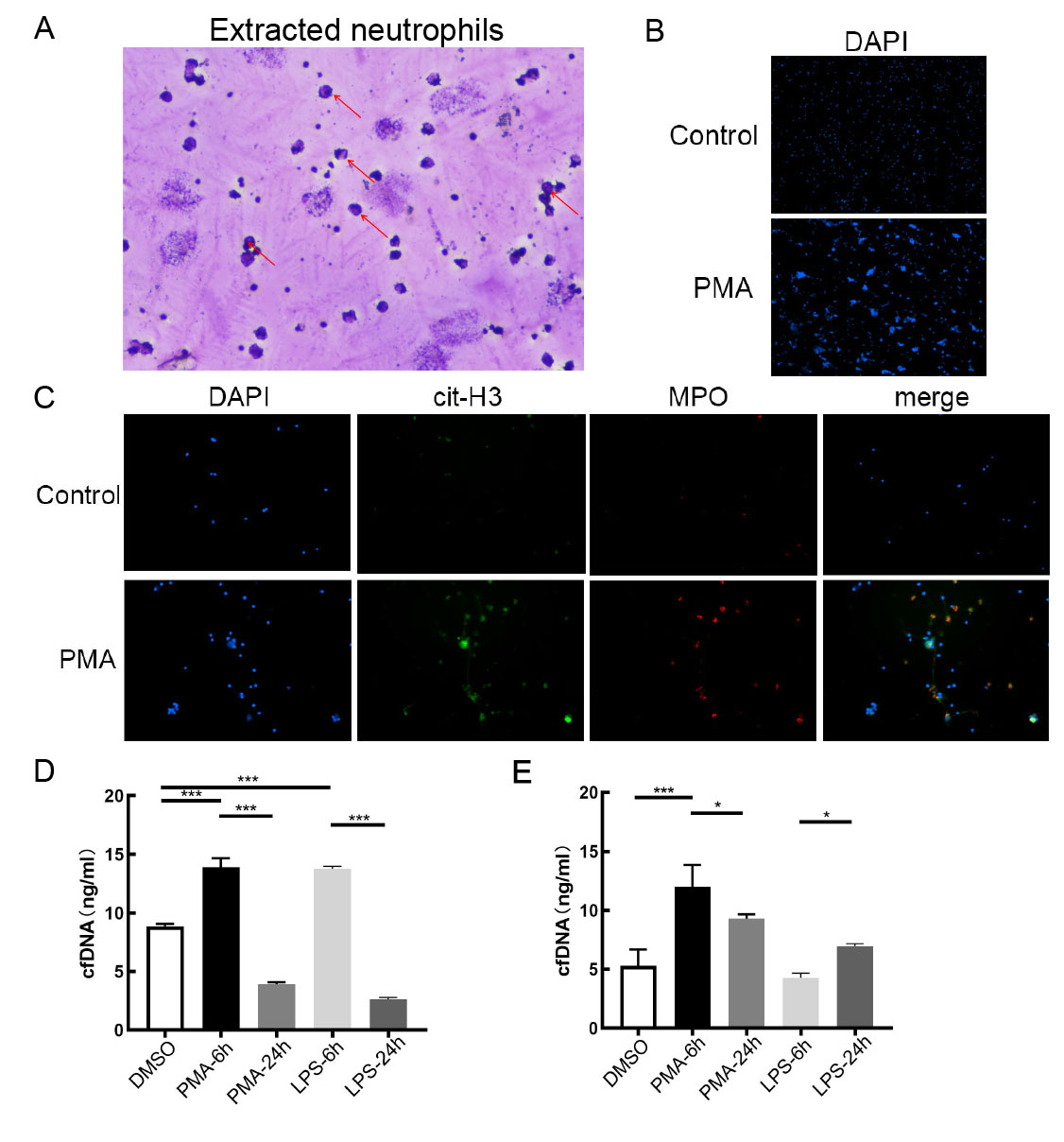

Supplement: Supplemental Information 17 — (A) Reichsen-Giemsa staining of neutrophils extracted. Arrows indicate cells with distinct lobed nuclei. (B) Field of view of DAPI stained neutrophils in Control group and PMA 6h-induction group. (4X objective magnification) (C) Immunofluorescence of NETs formation. (10X objective magnification) (D) cfDNA concentration of culture medium in control group(DMSO group), PMA 6h-induction group, PMA 24 h-induction group, LPS 6h-induction group and LPS 24h-induction group. (***, p < 0.001) (E) cfDNA concentration of washing culture medium in control group(DMSO group), PMA 6h-induction group, PMA 24h-induction group, LPS 6h-induction group and LPS 24h-induction group. (*, p < 0.05; ***, p < 0.001) [file peerj-12-16748-s017.jpg]

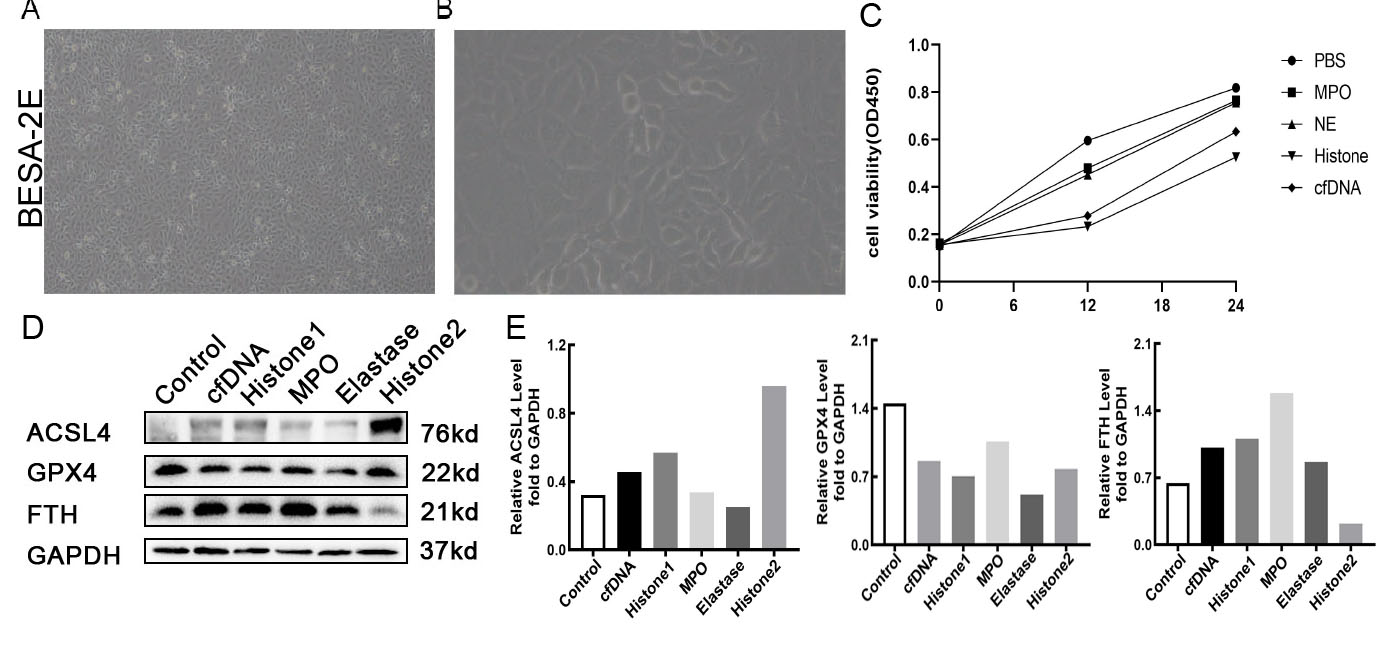

Supplement: Supplemental Information 18 — (A) Representative images of BESA-2E cells. (4X objective magnification) (B) Representative images of BESA-2E cells. (10X objective magnification) (C) Cell viability of BESA-2E cells after treatment of NETs components. (D) Western Blot of Ferroptosis marker protein expression of BESA-2E cells after treatment of NETs components for 6 h. Histone1 group was treated with 10 ug/ml histone for 6 h, Hisone2 group was treated with 10 ug/ml histone for 24 h. (E) Semi-quantitative figure of Western Blot. [file peerj-12-16748-s018.jpg]

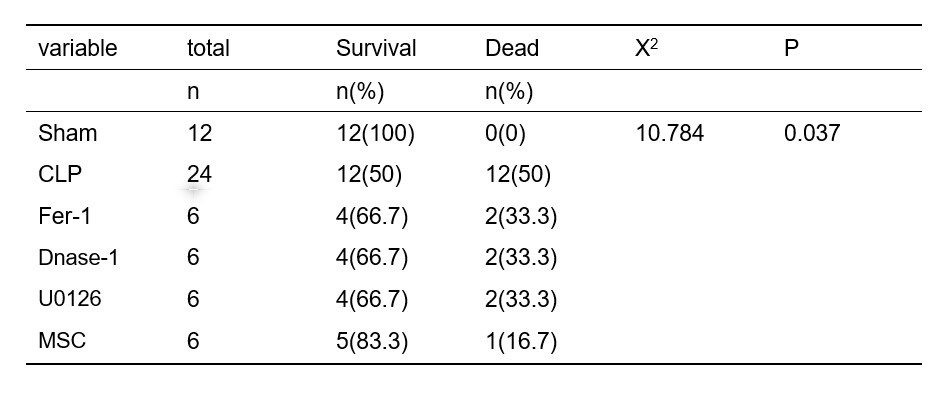

Supplement: Supplemental Information 22 [file peerj-12-16748-s022.jpg]

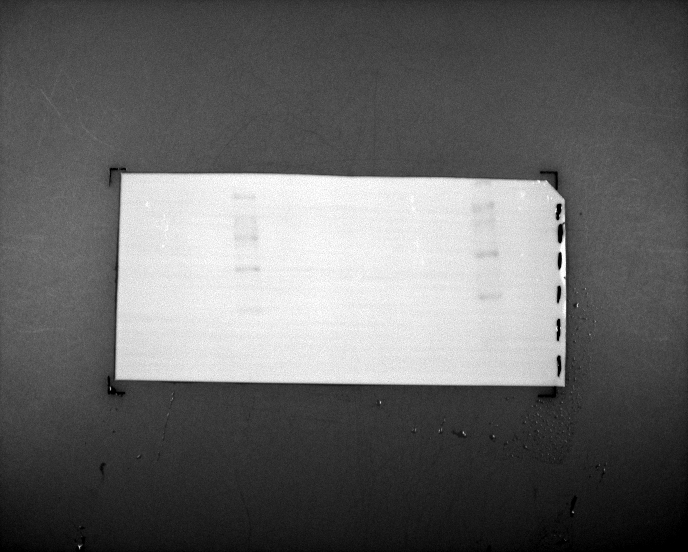

Supplement: Supplemental Information 23 [file peerj-12-16748-s023.zip › ╧╕░√╩╡╤Θú1⁄4WB/1.tif]

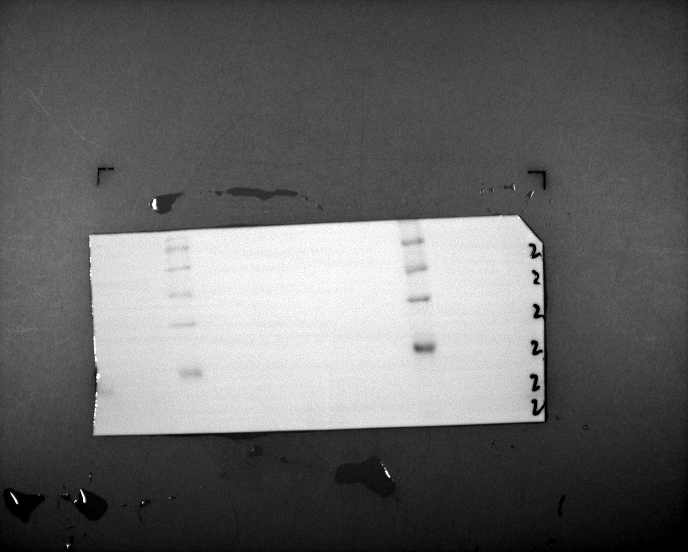

Supplement: Supplemental Information 23 [file peerj-12-16748-s023.zip › ╧╕░√╩╡╤Θú1⁄4WB/2.tif]

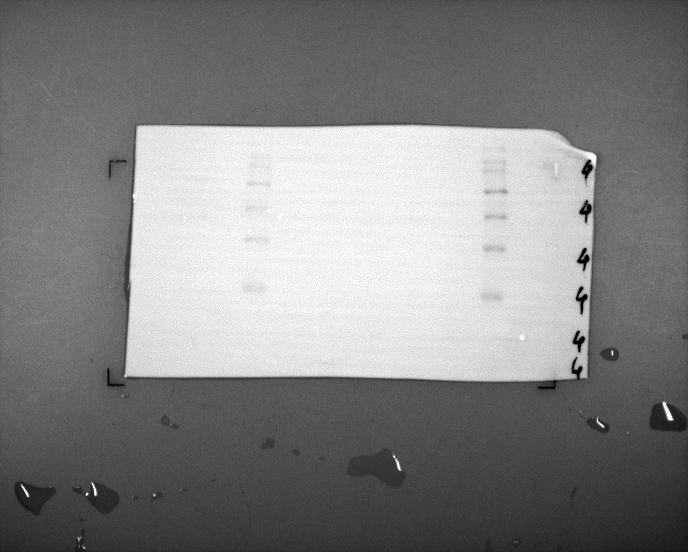

Supplement: Supplemental Information 23 [file peerj-12-16748-s023.zip › ╧╕░√╩╡╤Θú1⁄4WB/4.tif]

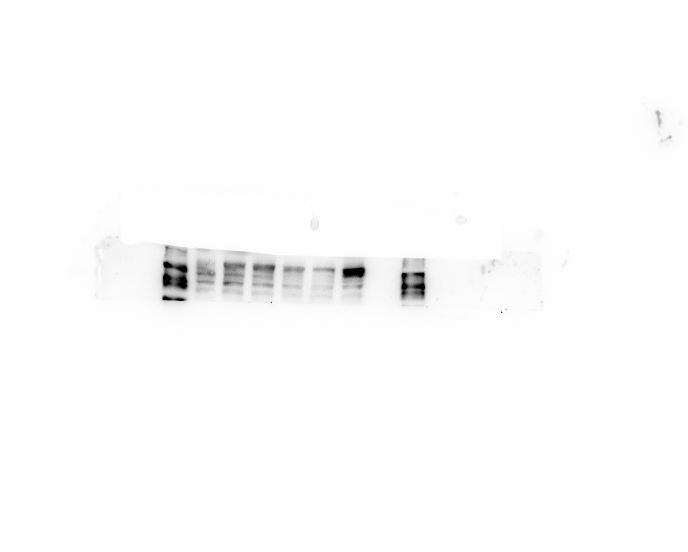

Supplement: Supplemental Information 23 [file peerj-12-16748-s023.zip › ╧╕░√╩╡╤Θú1⁄4WB/acsl41-1.tif]

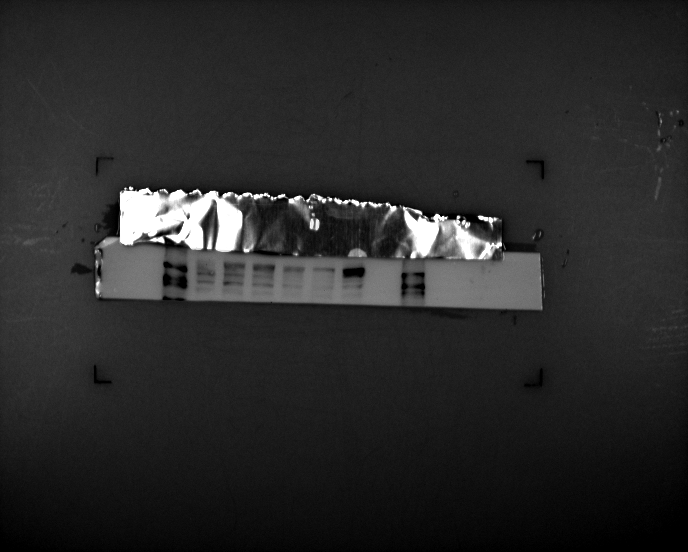

Supplement: Supplemental Information 23 [file peerj-12-16748-s023.zip › ╧╕░√╩╡╤Θú1⁄4WB/acsl41-2.tif]

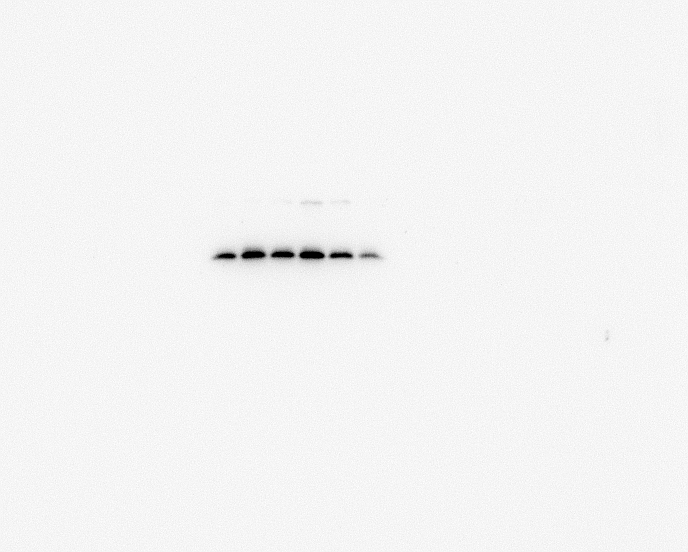

Supplement: Supplemental Information 23 [file peerj-12-16748-s023.zip › ╧╕░√╩╡╤Θú1⁄4WB/fth2-1.tif]

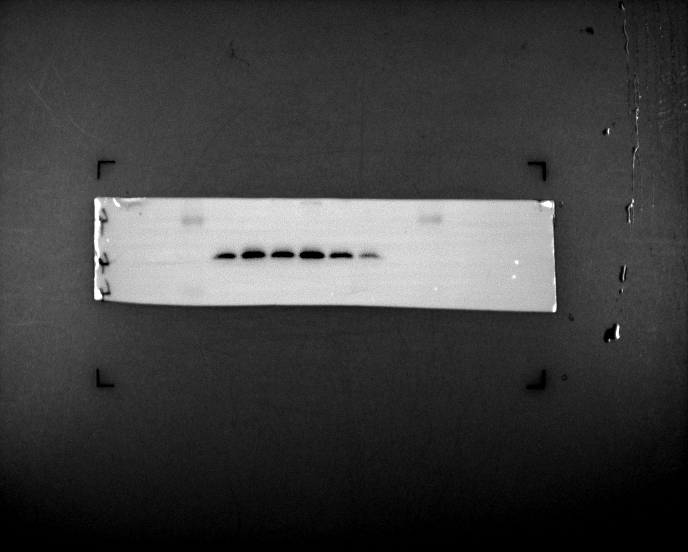

Supplement: Supplemental Information 23 [file peerj-12-16748-s023.zip › ╧╕░√╩╡╤Θú1⁄4WB/fth2-2.tif]

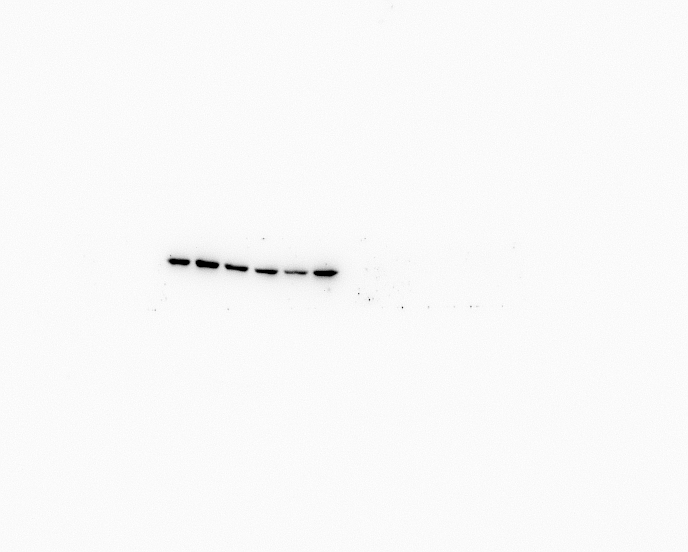

Supplement: Supplemental Information 23 [file peerj-12-16748-s023.zip › ╧╕░√╩╡╤Θú1⁄4WB/gapdh1-1.tif]

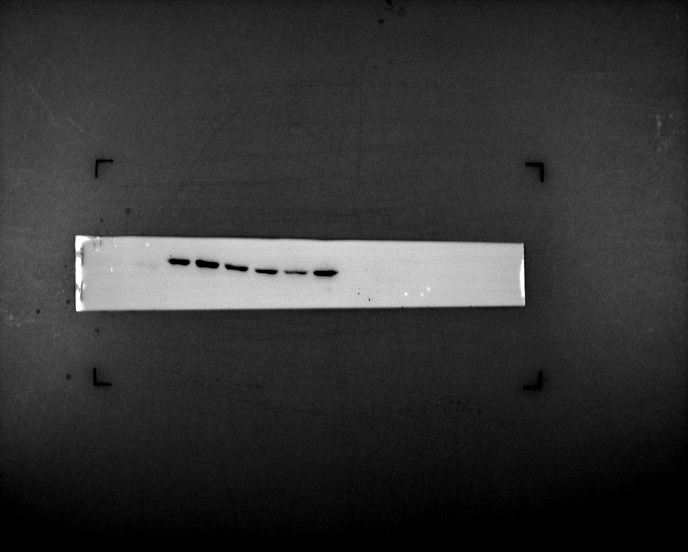

Supplement: Supplemental Information 23 [file peerj-12-16748-s023.zip › ╧╕░√╩╡╤Θú1⁄4WB/gapdh1-2.tif]

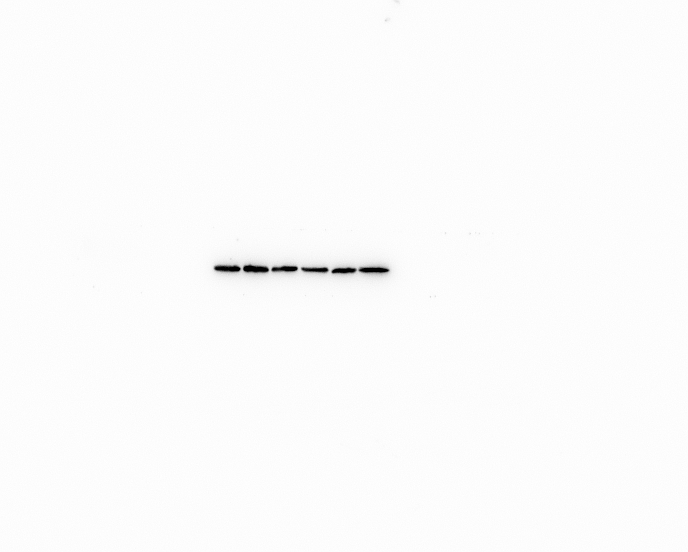

Supplement: Supplemental Information 23 [file peerj-12-16748-s023.zip › ╧╕░√╩╡╤Θú1⁄4WB/gapdh2-1.tif]

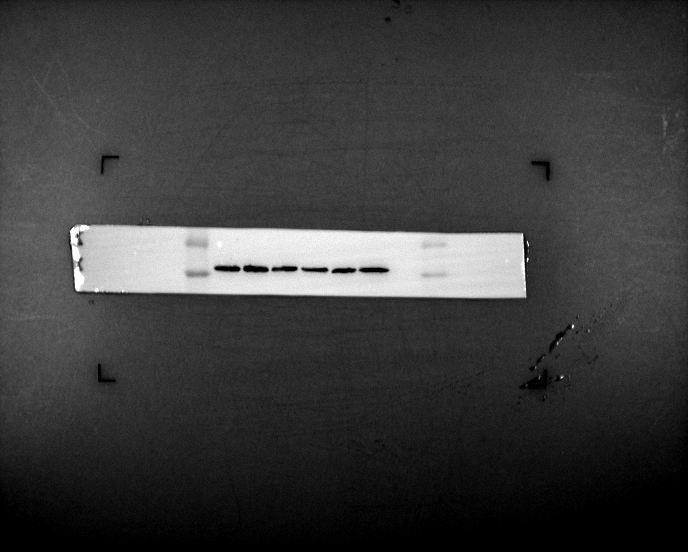

Supplement: Supplemental Information 23 [file peerj-12-16748-s023.zip › ╧╕░√╩╡╤Θú1⁄4WB/gapdh2-2.tif]
