# Supplementary figures and images for: Mesenchymal stem cells alleviate sepsis-induced acute lung injury by blocking neutrophil extracellular traps formation and inhibiting ferroptosis in rats (part 2 of 2)
Source: PeerJ. 2024 Jan 29;12:e16748. doi: 10.7717/peerj.16748 (PMC10832623; doi:10.7717/peerj.16748)

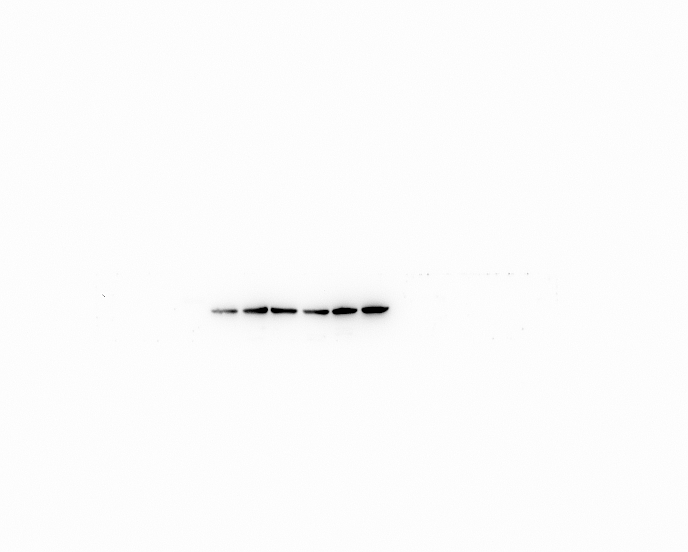

Supplement: Supplemental Information 23 [file peerj-12-16748-s023.zip › ╧╕░√╩╡╤Θú1⁄4WB/gapdh4-1.tif]

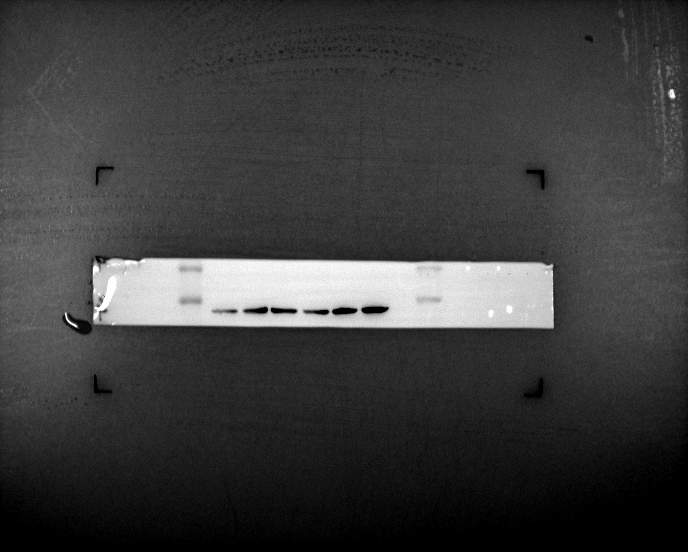

Supplement: Supplemental Information 23 [file peerj-12-16748-s023.zip › ╧╕░√╩╡╤Θú1⁄4WB/gapdh4-2.tif]

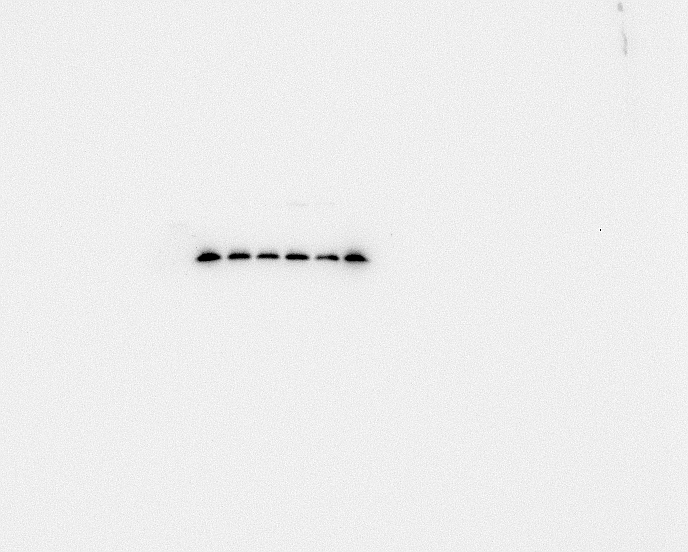

Supplement: Supplemental Information 23 [file peerj-12-16748-s023.zip › ╧╕░√╩╡╤Θú1⁄4WB/gpx4-3.tif]

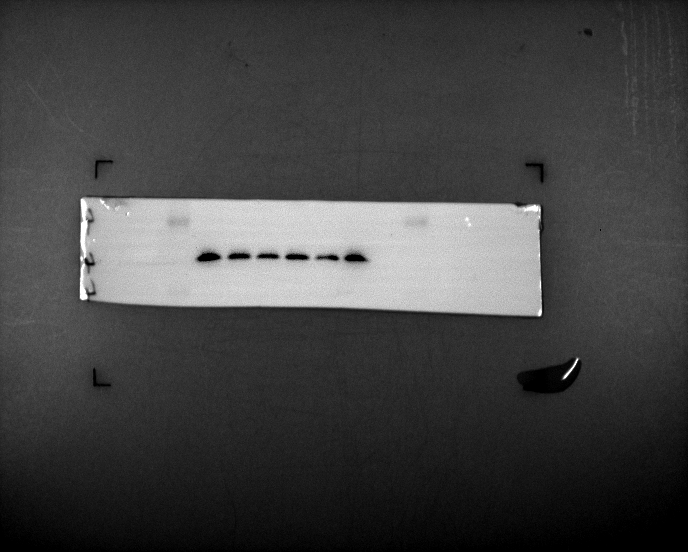

Supplement: Supplemental Information 23 [file peerj-12-16748-s023.zip › ╧╕░√╩╡╤Θú1⁄4WB/gpx4-4.tif]

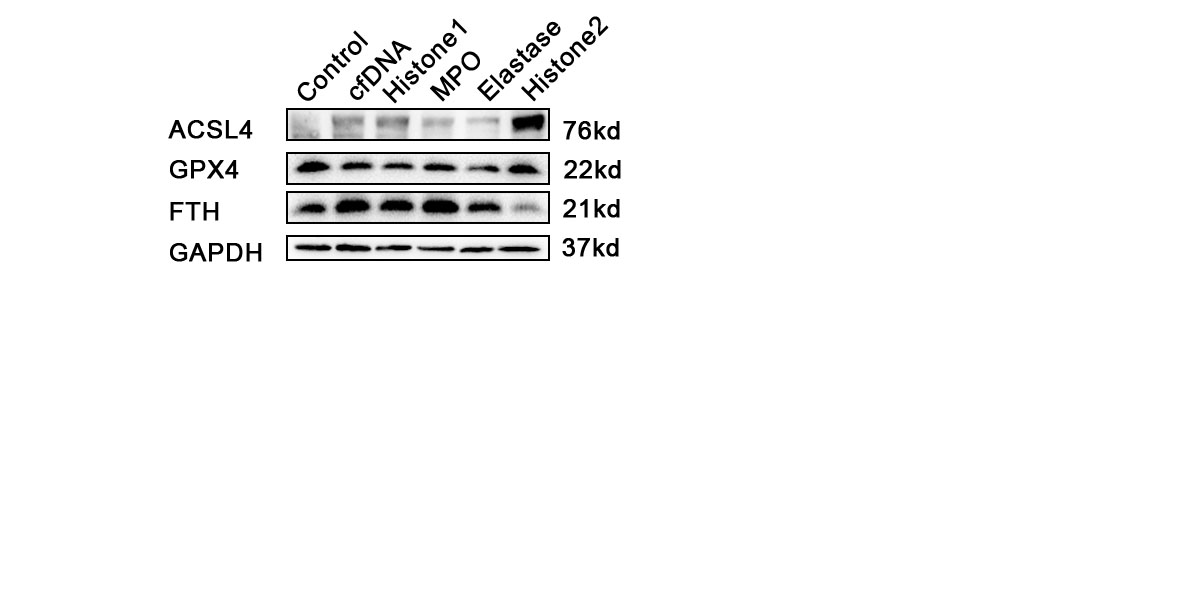

Supplement: Supplemental Information 23 [file peerj-12-16748-s023.zip › ╧╕░√╩╡╤Θú1⁄4WB/WB-cell.jpg]
