# Supplementary material for: Mesenchymal stem cells alleviate sepsis-induced acute lung injury by blocking neutrophil extracellular traps formation and inhibiting ferroptosis in rats
Source: PeerJ. 2024 Jan 29;12:e16748. doi: 10.7717/peerj.16748 (PMC10832623; doi:10.7717/peerj.16748)
Supplement: Supplemental Information 24 [file peerj-12-16748-s024.docx]

Death rate of our CLP modeling was 50%, lower than death rate of Sham group. Treatment had a certain reduction in mortality compared with CLP group. MSC group had the lowest mortality in our experiment, but there was no statistical difference compared with the inhibitor group (Supplementary Table 1).


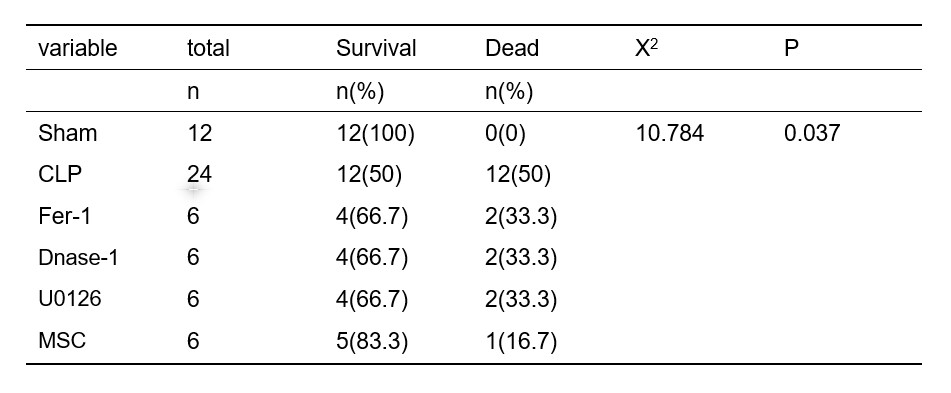


**Supplementary Table 1. Survival and Death rate of animal modeling**

Fisher's exact test results of multi-group Chi-square test was used to detect statistical analysis.

ACSL4 expression in CLP was higher than Sham group. Using of Fer-1, Dnase-1, U0126 and MSC could reverse this tendency. (Supplementary 1A-1E). NETs information improved in CLP group, compared with Sham group. Dnase-1 could totally reduce NETs formation and Fer-1 only reduced NETs formation (Supplementary 1F).


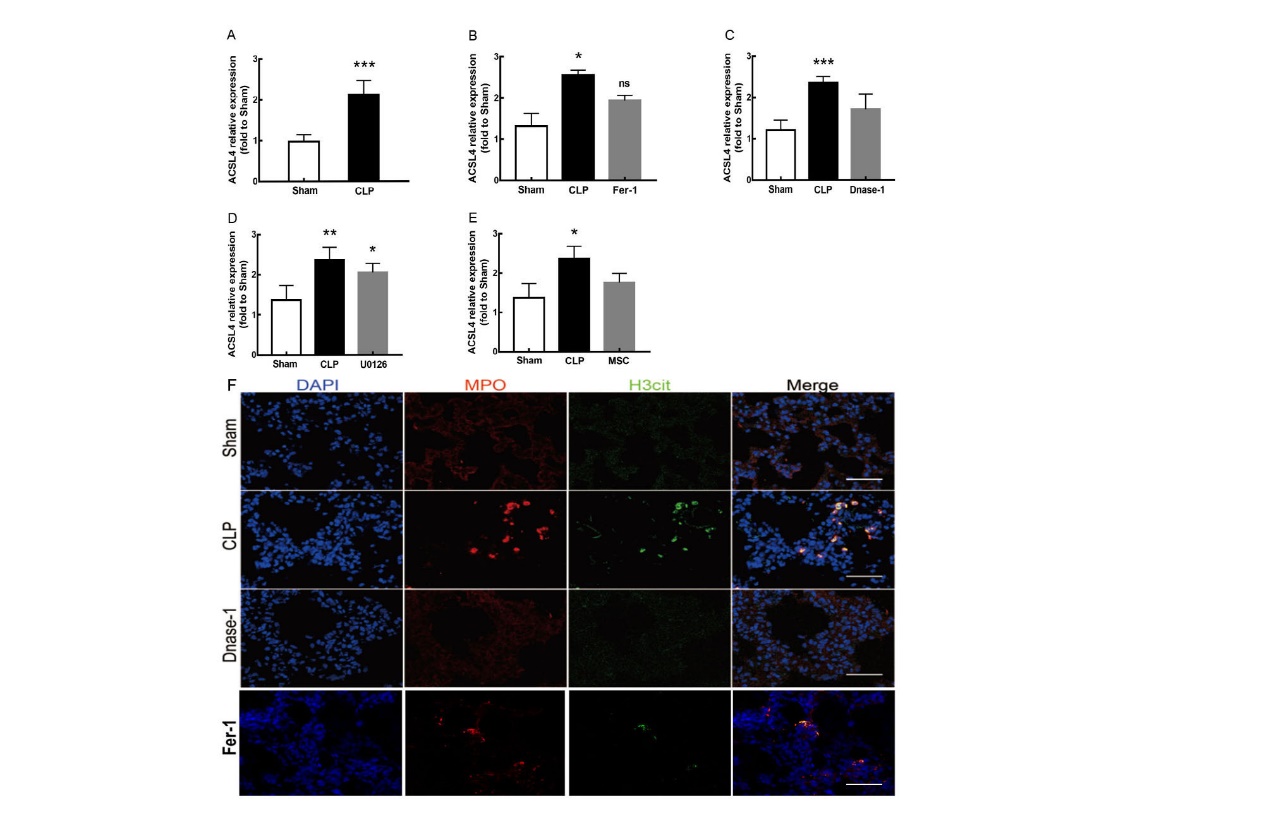


**Supplementary 1. Extra data of figures in manuscript** (A) Grayscale statistic of immunohistochemistry picture in figure 1. (*compared with Sham group, ***, p＜0.001) (B) Grayscale statistic of immunohistochemistry picture in figure 2. (*, p＜0.05; ns, no statistical significance) (C) Grayscale statistic of immunohistochemistry picture in figure 3. (***, p＜0.001) (D) Grayscale statistic of immunohistochemistry picture in figure 5 (*, p＜0.05; **, p<0.01) (E) Grayscale statistic of immunohistochemistry picture in figure 7 (*, p＜0.05) (F) Nets formation in Sham, CLP, Dnase-1, Fer-1 group.

Human blood neutrophils were isolated by neutrophil isolation solution kit kit. (Supplementary 2A) cfDNA had been reported as a correlation factor of high-mobility in sepsis(Cheng et al. 2020). Phorbol 12-myristate 13-acetate (PMA) was reported as NETs induction(Najmeh et al. 2015). Lipopolysaccharides (LPS) was an important damaging factor in sepsis and was also been reported as an inducer of NETs(Haute et al. 2015). We set up five group as PMA 6h-induction group, PMA 24h-induction group, LPS 6h-induction group and LPS 24h-induction group and Control group, which was cultured with same concentration of DMSO as induction groups. After PMA induction, NETs formation was improved. (Supplementary 2B-C). We found that cfDNA concentration in PMA-6h and LPS-6h group was significantly higher than that in Control group, and cfDNA concentration in the 6h induction group was higher than that in the 24-hour group (Supplementary 2D). Culture medium contained PMA/LPS components, might affect subsequent experiments. We detected cfDNA concentration in wash culture medium and found PMA-6h group have the highest concentration of cfDNA (Supplementary 2E). Compared with culture medium, concentration of cfDNA in wash culture medium was similar after induction (Supplementary 2D-E).


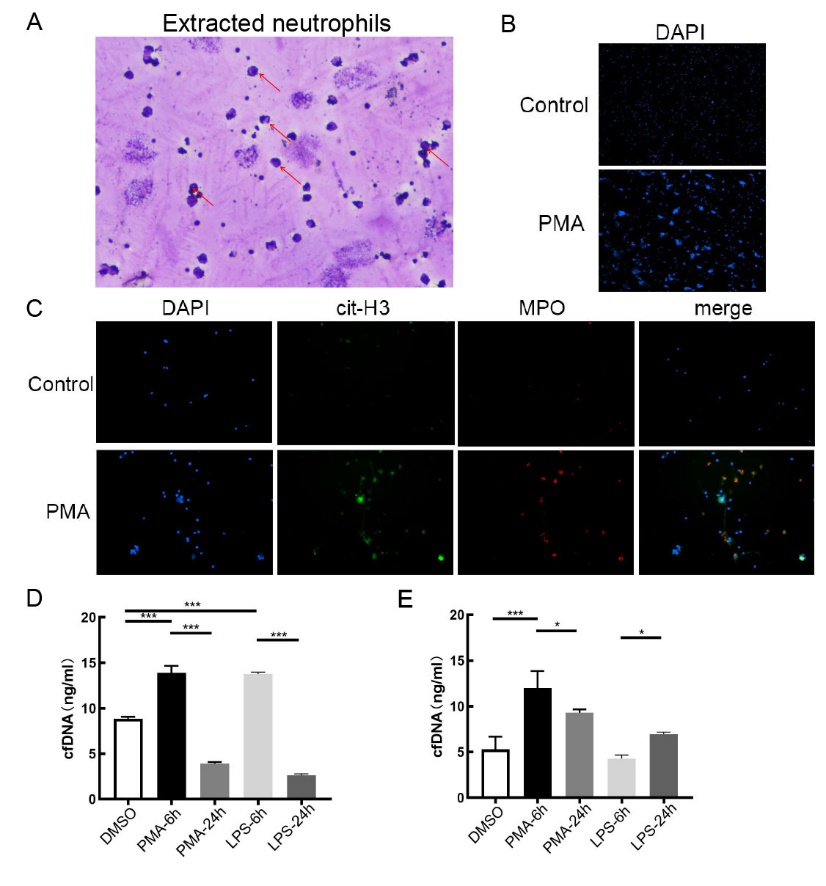


**Supplementary 2. Acquisition of cfDNA from NETs induced by human blood neutrophils in vitro** (A) Reichsen-Giemsa staining of neutrophils extracted. Arrows indicate cells with distinct lobed nuclei. (B) Field of view of DAPI stained neutrophils in Control group and PMA 6h-induction group. (4X objective magnification) (C) Immunofluorescence of NETs formation. (10X objective magnification) (D) cfDNA concentration of culture medium in control group(DMSO group), PMA 6h-induction group, PMA 24h-induction group, LPS 6h-induction group and LPS 24h-induction group. (***, p<0.001) (E) cfDNA concentration of washing culture medium in control group(DMSO group), PMA 6h-induction group, PMA 24h-induction group, LPS 6h-induction group and LPS 24h-induction group. (*, p<0.05; ***, p<0.001)

It was reported that alveolar epithelium and lung epithelial cells were the main injury cells in the occurrence of lung injury(Kumar 2020; Tao et al. 2023). We got lung epithelial cells to further experiment. (Supplementary 3A-B) We examined the effects of NETs components on the activity of BESA-2E cells. Histone and cfDNA had greater effect on cell activity (Supplementary 3C). Then, we examined ACSL4, GPX4 and FTH level in BESA-2E cells after NETs components treatment. ACSL4 and FTH expression was increased with cfDNA or histone treatment, but longer histone treatment seemed to decrease FTH expression. GPX4 expression decreased with all NETs components treatment (Supplementary 3D-E).


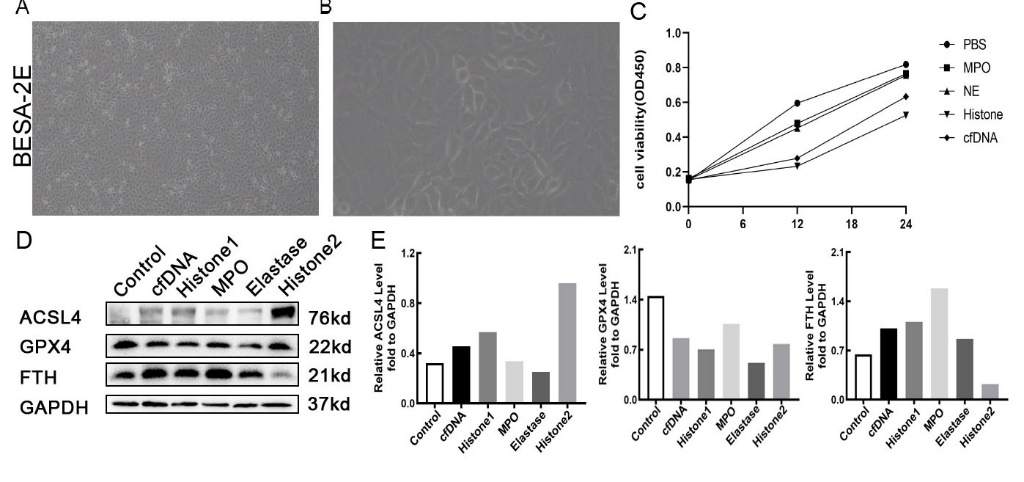


**Supplementary 3. Effects of NETs components on lung epithelial cells** (A) Representative images of BESA-2E cells. (4X objective magnification) (B) Representative images of BESA-2E cells. (10X objective magnification) (C) Cell viability of BESA-2E cells after treatment of NETs components. (D) Western Blot of Ferroptosis marker protein expression of BESA-2E cells after treatment of NETs components for 6h. Histone1 group was treated with 10ug/ml histone for 6h, Hisone2 group was treated with 10ug/ml histone for 24h. (E) Semi-quantitative figure of Western Blot.

Methods:

1.Immunohistochemical gray density analysis

We adjusted the contrast to make background in all immunohistochemical picture from the same figure to white (gray density=0). Then, we adjusted images’ RGB stack threshold in image j software to transform the immunohistochemistry from color images to grayscale images, set the frame selection threshold to make the selection area include and only include the region with obvious brown color in the immunohistochemistry. Finally, we detected the grayscale value of the selection area. The difference in the expression of ACSL4 protein was demonstrated by the ratio of each group in the same set of images compared with the negative control.

2. Immunohistochemistry and Immunofluorescence

The same as Methods 2.8 and Methods 2.9 in our manuscript.

3. Neutrophils extraction

We use Human neutrophil isolation solution kit (Tianjin Haoyang Biology Manufacture Co.,Ltd, Tianjin China, LZS11131) to extract neutrophils. Human peripheral venous blood was provided by healthy adult volunteers and was isolated and cultured immediately after blood collection. 3mL of EDTA anticoagulant whole blood was gently superimposed on the surface of 5mL of neutrophil isolation solution, centrifuged at 600g for 30min. Six layers of cells would appear from top to bottom in the centrifuge tube, the first layer was diluted plasma, the second and fourth layers were ring opalactic cell layers, the second layer was mononuclear cell layer, the fourth layer was neutrophil layer, and the fifth layer was isolation solution. Layer 6 is the red blood cell layer. The fourth layer of neutrophils was absorbed and transferred to another centrifuge tube. Add 10mL cleaning solution, mix well, centrifuge 300g for 10min, discard the supernatant; Appropriate amount of red blood cell lysate was added, lysate at 4℃ for 10min, centrifuge at 300g for 10min, discard the red supernatant, and repeat lysate if incomplete lysate was found. The precipitated cells were re-suspended with 5mL of cleaning solution using a straw, centrifuged at 300g for 10min, and the supernatant was abandoned. The precipitated cells were re-suspended again, centrifuged at 300g for 10min, and the supernatant was abandoned, and the cells were re-suspended with 3mL of RPMI 1640 culture medium (GIBCO, China, C11875500BT) containing 10% fetal bovine serum (GIBCO, Australia, 10099141), and cultured at 37 ℃, 95% humidity, and 5% CO2.

4. Reichsen-Giemsa staining of neutrophils extracted

We used reagent provided in Human neutrophil isolation solution kit (Tianjin Haoyang Biology Manufacture Co.,Ltd, Tianjin China, LZS11131) to conduct Reichsen-Giemsa staining. A drop of cell suspension was added to the middle of the slide, dried. Then, adding 3-5 drops of reagent I, keep the slide wet, stain for 30s, then add reagent II, which is more than twice the volume of reagent I, mix well, stain for 5-8min, and then rinse the slide slowly with clean water or soak the slide for 10-15min. After the slides were dried, the morphology of neutrophils was observed under an optical microscope. Four cells in the field of vision were counted under a 200-fold microscope, and the ratio of neutrophils to the total number of cells under the microscope was simply calculated to calculate the purity of the isolated neutrophils.

5. NETs formation induced in vitro

Cells were inoculated on a 24-well plate treated with polylysine at a rate of 2-5×105 cells per well, each well was 500μL, and the 24-well plate was cultured in a CO2 incubator at 37℃ with 95% humidity and 5% for 1h. Then they were given 10nmol/l PMA (Acmec, Shanghai China, P33390), 5ug/ml LPS (Sigma-Aldrich, America, L2880) stimulation. Both PMA and LPS were dissolved with DMSO and then diluted with neutrophil culture solution. Control group was cultured at the same concentration of DMSO as PMA/LPS reagent. For PMA/LPS induction group, we set up 6h and 24h induction groups respectively.

6.Cell-free DNA(cfDNA) extraction and concentration detection

To detect the level of NETs induced by PMA and LPS, we chose to detect the DNA concentration after induction. DNA levels in culture medium was detected at corresponding time point. At the same time, we removed culture medium and washed down cfDNA remained at Petri dish with RPMI 1640 culture medium containing 10% fetal bovine serum for 3 times. After that we gain supernatant centrifugated with 1000 g, and centrifuged at 4℃ for 5 min and detected DNA concentration in the supernatant. DNA at the corresponding time Purification Kit (Beyotime, China, D0033) was used to extract cfDNA. According to the protocol of kit, cfDNA was obtained by DNA purification column. Finally, purified cfDNA was obtained by dissolution in 50ul ddH2O. The DNA concentration was detected by Nanodrop One ultramicro spectrophotometer (Gene Company Limited, China).

7. Cell culture and experiment design

Human Lung epithelial (BESA-2E) cells were obtained from ATCC (America, CRL-3588), and cultured in RPMI 1640 culture medium (GIBCO, China, C11875500BT) containing 10% fetal bovine serum (GIBCO, Australia, 10099141), and cultured at 37 ℃, 95% humidity, and 5% CO2. In order to study which components of NETs are the main causes of lung epithelial cell injury, we selected the most common elements like cfDNA, histone, myeloperoxidase (MPO), and elastase in NETs. 5ng/ml cfDNA (extracted with the method described earlier), 10ug/ml histone (Sigma, China, H9250), 10ug/ml MPO (Med Chem Express, China, HY-P70255A), 10ug/ml elastase (Med Chem Express, China, HY-P2974) were used to culture cells for 6h.

8.Cell viability

BESA-2E cell viability after treatment with different component of NETs was determined using the cell counting kit 8(CCK-8) （Biosharp, China, BS350B）. Briefly, the treated cells were collected, and the culture was converted to 10% CCK-8 fresh medium. The absorbance at 450 nm was determined using a multimode microplate reader.

9. Immunoblotting.

The same as Methods 2.10 in our manuscript.

10. Statistical Analysis

Each biological experiment was performed in at least three replicates. Results were expressed as mean ±SEM. Differences between two groups were analyzed by indepentent samples t test and between above two groups were analyzed by one-way analysis of variance. Turkey's test is used for further comparison. GraphPad Prism 6 software was used for statistical analysis of all experimental data, and p < 0.05 was used as the threshold for statistical significant differences.

Cheng Z, Abrams ST, Toh J, Wang SS, Wang Z, Yu Q, Yu W, Toh CH, and Wang G. 2020. The Critical Roles and Mechanisms of Immune Cell Death in Sepsis. *Front Immunol* 11:1918. 10.3389/fimmu.2020.01918

Haute GV, Caberlon E, Squizani E, de Mesquita FC, Pedrazza L, Martha BA, da Silva Melo DA, Cassel E, Czepielewski RS, Bitencourt S, Goettert MI, and de Oliveira JR. 2015. Gallic acid reduces the effect of LPS on apoptosis and inhibits the formation of neutrophil extracellular traps. *Toxicol In Vitro* 30:309-317. 10.1016/j.tiv.2015.10.005

Kumar V. 2020. Pulmonary Innate Immune Response Determines the Outcome of Inflammation During Pneumonia and Sepsis-Associated Acute Lung Injury. *Front Immunol* 11:1722. 10.3389/fimmu.2020.01722

Najmeh S, Cools-Lartigue J, Giannias B, Spicer J, and Ferri LE. 2015. Simplified Human Neutrophil Extracellular Traps (NETs) Isolation and Handling. *J Vis Exp*. 10.3791/52687

Tao H, Xu Y, and Zhang S. 2023. The Role of Macrophages and Alveolar Epithelial Cells in the Development of ARDS. *Inflammation* 46:47-55. 10.1007/s10753-022-01726-w
